# Diversity and structure of sparids external microbiota (Teleostei) and its link with monogenean ectoparasites

**DOI:** 10.1186/s42523-022-00180-1

**Published:** 2022-04-13

**Authors:** Mathilde Scheifler, Sophie Sanchez-Brosseau, Elodie Magnanou, Yves Desdevises

**Affiliations:** Biologie Intégrative des Organismes Marins, BIOM, Observatoire Océanologique, Sorbonne Université - CNRS, 66650 Banyuls/Mer, France

**Keywords:** Sparidae, Microbiota, Phylosymbiosis, Metabarcoding, Parasite, Monogenea, *Lamellodiscus*, Tripartite interaction

## Abstract

**Background:**

Animal-associated microbial communities appear to be key factors in host physiology, ecology, evolution and its interactions with the surrounding environment. Teleost fish have received relatively little attention in the study of surface-associated microbiota. Besides the important role of microbiota in homeostasis and infection prevention, a few recent studies have shown that fish mucus microbiota may interact with and attract some specific parasitic species. However, our understanding of external microbial assemblages, in particular regarding the factors that determine their composition and potential interactions with parasites, is still limited. This is the objective of the present study that focuses on a well-known fish-parasite interaction, involving the Sparidae (Teleostei), and their specific monogenean ectoparasites of the *Lamellodiscus* genus. We characterized the skin and gill mucus bacterial communities using a 16S rRNA amplicon sequencing, tested how fish ecological traits and host evolutionary history are related to external microbiota, and assessed if some microbial taxa are related to some *Lamellodiscus* species.

**Results:**

Our results revealed significant differences between skin and gill microbiota in terms of diversity and structure, and that sparids establish and maintain tissue and species-specific bacterial communities despite continuous exposure to water. No phylosymbiosis pattern was detected for either gill or skin microbiota, suggesting that other host-related and environmental factors are a better regulator of host-microbiota interactions. Diversity and structure of external microbiota were explained by host traits: host species, diet and body part. Numerous correlations between the abundance of given bacterial genera and the abundance of given *Lamellodiscus* species have been found in gill mucus, including species-specific associations. We also found that the external microbiota of the only unparasitized sparid species in this study, *Boops boops*, harbored significantly more *Fusobacteria* and three genera, *Shewenella*, *Cetobacterium* and *Vibrio*, compared to the other sparid species, suggesting their potential involvement in preventing monogenean infection.

**Conclusions:**

This study is the first to explore the diversity and structure of skin and gill microbiota from a wild fish family and present novel evidence on the links between gill microbiota and monogenean species in diversity and abundance, paving the way for further studies on understanding host-microbiota-parasite interactions.

**Supplementary Information:**

The online version contains supplementary material available at 10.1186/s42523-022-00180-1.

## Background

Teleost fish include more than 28,000 species, which represent a broad range of physiologies, ecologies and natural histories [1]. From their skin surface to their gastrointestinal tract, teleost fish harbor a large diversity of symbiotic macro- and microorganisms, including eukaryotes and bacteria that can be pathogenic (such as parasites) or not [2–5]. Teleost fish therefore represent a suitable vertebrate group for understanding the composition of endo- and ectosymbiotic communities, as well as the endogenous and exogenous factors and mechanisms shaping these symbiotic relationships [6].

The external surface of animals is an important primary barrier, particularly in fish, surrounded by highly abundant and diverse organisms (viruses, bacteria, archaea and eukaryotes) present in the water [7, 8]. External mucus is a viscous substance covering the skin and gills of teleost fish [9]. The microbial communities present in this mucus have been shown to be of primary importance in the relationship of teleosts with their biotic and abiotic environment regarding host ecology (choice of sexual partner, embryonic development, social behavior) [10, 11], but also host fitness as they have been shown to enhance nutrient absorption, modulate the immune system and protect against pathogens [12–16].

To date, most studies on fish microbiota have investigated fish gut communities but progress has been made in recent years concerning gill mucus and especially skin mucus microbiota. A number of environmental factors have been suggested to shift the composition of skin mucus microbiota, such as salinity [17], seasonality or temperature [18–20]. One part of these variations may be due to the timing of plankton blooms and changes in the microbial community from the surrounding water [21]. External microbiota is also distinct between fish species [22–24] and these differences can be related to host ecological traits [23]. A pattern called “phylosymbiosis” has been recently reported for skin microbiota of coral reef fish [23]. The concept of phylosymbiosis describes the eco-evolutionary pattern that occurs when similarities observed between host microbiota mirror host evolutionary relationships [25]. Therefore, hosts that are more phylogenetically related will harbor microbial communities more ecologically similar. This pattern can occur via several mechanisms, including vertical transmission of bacterial lineages across host generations and/or coevolution of microbial communities with their host [25, 26]. It can be also inferred if closely related hosts with similar genetic or behavioral and ecological traits select similar bacterial lineages from the external environment [25–27]. However, despite their importance in conserving homeostasis and preventing infection, skin and gill mucus microbiota diversity remains poorly known with the exception of a few captive species used in aquaculture, species of commercial interest or model organisms such as zebrafish [28, 29]. More specifically, there is little knowledge about the factors (endogenous or exogenous) explaining the diversity and variability of skin and gill mucus microbiota of wild teleost fish.

External fish mucus microbiota plays an essential role in the immunity against pathogens but at the same time attracts and harbors specific parasitic species [30]. Fish gills and skin are well known to be parasitized by many eukaryotic organisms, unicellular or multicellular [31]. Monogeneans (Platyhelminthes) are very common ectoparasites, often abundant on the fish skin and gills. They are generally highly host-specific, i.e. a parasitic species infects only one or a few host species [31, 32]. Adult monogeneans lay eggs in the water column that hatch into ciliated larvae (oncomiracidia) that are attracted to the mucus of teleost fish species [33, 34]. When reaching the host, the larvae lose their ciliature, some larvae remain on the skin but most migrate from the skin to the gills of the fish to develop in adults. Several studies propose that the specificity of monogeneans is governed by factors present in the external mucus [35–38]. However, the mechanisms of establishment and the determinants of the specificity and attraction of monogeneans to their hosts remain poorly known [39]. Recent work suggested that external bacterial communities have a role in the production of chemical stimuli that interact with monogenean larvae [40, 41]. Increasing evidence supports close interaction between parasitic species and fish microbiota, which in turn influences host physiology [42–44]. These recent studies highlighted that the presence of parasitic species, the intensity of infection or the abundance of parasites was highly correlated with particular microbial taxa [45–47]. However, most of these studies investigated gut microbial communities of fish and their link with intestinal parasites (mainly nematodes and cestodes), and there is currently very little knowledge about the interaction between skin or gill mucus microbiota of fish and their ectoparasites, such as monogeneans, especially in natural populations [40].

The present study tackled a well-known fish-parasite system in the Mediterranean Sea: the association between Sparidae (Perciformes) and their specific monogenean gill ectoparasites belonging to the *Lamellodiscus* genus. Sparids include 19 species in the north-eastern Mediterranean Sea that present contrasting life histories (diet, ecology, life style) (Fishbase, www.fishbase.se). The host specificity of each *Lamellodiscus* species, i.e. the number of hosts per parasite species, has been intensively studied in Mediterranean sparids and can thus be considered as well-known [48–62] (Additional file 1: Table S1). Surprisingly, two Mediterranean sparid species were never found to be parasitized by *Lamellodiscus*, *Boops boops* and *Dentex dentex*. The aim of this study was to characterize the microbial communities living within the external mucus (both skin and gills) of north-Mediterranean sparids and assess the effect of environmental factors, as well as host’s ecological traits and evolution, on the structure and diversity of these two microbiota. We then characterized the composition and abundances of ectoparasitic *Lamellodiscus* gills communities in each fish species in order to assess if they are related to abundances of given microbial taxa within the mucus. It allowed us to understand how the interaction between microbial communities and parasites can be related to monogenean host specificity.

## Materials and methods

### Sampling

Fish sampling was conducted between June 2017 and July 2019 in the Bay of Banyuls-sur-Mer (northwest Mediterranean Sea, France) (Table 1). For all fish individuals, a gill fishing net was placed overnight between 0 and 6 m depth. About 6 h later, fish were collected from the net, handled with gloves and put into individual plastic bags right after collection. They were immediately brought from the vessel to the laboratory for dissection. Skin mucus and gill mucus (by taking the first gill arch) were collected per fish individual with a sterile spatula and scissors. For all collected fish, skin mucus was scraped off with a sterile spatula from the central part of the body to above the lateral line on both sides of the fish. We also collected the same gill arch from all fish individuals, while the seven other arches were used to determine *Lamellodiscus* species diversity and abundance. Between each sample, scissors and spatula were sterilized. Samples were immediately put into sterile tubes and frozen at − 80 °C until DNA extraction. A total of 62 fish individuals were sampled for their skin and gill mucus. They all belonged to 15 sparid species, 12 of them represented by at least 3 individuals and 3 of them represented by a single or 2 individuals (Table 1). Unfortunately, 4 sparid species were not sampled (*Diplodus cervinus*, *Lithognathus mormyrus*, *Spicara smaris* and *Spicara flexuosa*). The genus *Spicara* belongs to the family Sparidae based on phylogenetic analyses [63].

**Table 1:** Total number of DNA samples sequenced

Fish species	Number of individuals	Number of samples	
		Gill mucus	Skin mucus
*Boops boops*^2^	5	5	5
*Dentex dentex*^3^	1	1	1
*Diplodus annularis*^5^	5	5	5
*Diplodus puntazzo*^12^	1	1	1
*Diplodus sargus*^10,12,13^	6	6	6
*Diplodus vulgaris*^6,8^	5	5	4
*Oblada melanura*^6^	5	5	4
*Pagellus acarne*^5,9^	5	5	5
*Pagellus bogaraveo*^1^	4	4	4
*Pagellus erythrinus*^5,6,9^	5	5	4
*Pagrus pagrus*^2,4^	3	3	0
*Sarpa salpa*^8^	5	5	5
*Sparus aurata*^11^	5	5	5
*Spicara maena*^7^	5	5	3
*Spondyliosoma cantharus*^2,5^	2	2	1
**Subtotal**	62	62	53
Water samples	11		
**Total number of samples**	126		

Seawater samples have also been collected at each sampling date

Sampling in 2017: ^1^21 June

Sampling in 2018: ^2^18 April; ^3^26 June; ^4^13 July; ^5^4 September; ^6^5 October

Sampling in 2019: ^7^22 March; ^8^28 March; ^9^10 May; ^10^6 June; ^11^28 June; ^12^8 July; ^13^10 July

To assess the diversity and composition of microbial communities, seawater was also collected using a sterile container at each sampling date next to the gill net fishing. Two liters of seawater were filtered immediately after the fish sampling onto a 0.2 µm nitrocellulose filter (Pall Corporation, U.S.A). Filters were frozen in sterile cryotubes at − 80 °C until DNA extraction. The DNA of two surface water samples could not be amplified during subsequent steps and these samples were removed, making a total of 11 water samples included in this study (Table 1).

### Characterization of gill parasites

*Lamellodiscus* individuals were sampled from the seven other gill arches for each fish individual under a dissecting microscope. Counts and identification of *Lamellodiscus* specimens could not be carried on *Pagellus bogaraveo*, because these host individuals were collected in 2017 in another study that focused only on the characterization of the microbiota [64], therefore gill arches were not conserved. To assess the diversity and abundance of *Lamellodiscus* communities, we identified each individual based on the haptor and copulatory organ morphology under an optical microscope [48–58].

### DNA extraction and 16S rRNA amplification

DNA was extracted from 150 mg of skin or gill mucus by using the Quick-DNA Fecal/Soil Microbe MiniPrep Kit (Zymo Research, Orange, California) following manufacturer’s instructions and eluted in 50 µl of elution buffer. Samples were frozen at − 80 °C. PCR amplification was carried out in triplicate and performed using primers targeting the hypervariable V3-V4 region of the 16S rRNA gene: 341F (5’CCTACGGGNGGCWGCAG-3′) and 805R (5′-GACTACHVGGGTATCTAATCC-3′) [65, 66]. The PCR mix contained 5 µl of 1X KAPA 2G Fast Ready Mix (Sigma-Aldrich, France), 0.2 µl of each primer (concentration of 0.2 µM), 3.6 µl of ultrapure water and 1 µl of DNA in a final volume of 10 µl. After 3 min of initial denaturation at 95 °C, the following conditions were applied: 30 cycles of 95 °C for 45 s (denaturation), 50 °C for 45 s (annealing) and 68 °C for 90 s (extension), with a final extension at 68 °C for 5 min. For each sample, three PCRs were performed in the same conditions, to increase the DNA quantity and also to avoid bias due to each PCR reaction. Then, each PCR product was run on 1% agarose gel at 100 V for 20 min in an electrophoresis chamber (Mupid®-One) to visualize the presence of high molecular weight DNA. The visualization was carried out in a GelMaxTM photodocumenter (UVP®). When the DNA was visible in the gel, amplifications from the same sample were pooled. Individual barcode sequences were added to each mix during a second PCR. The second PCR mix contained 12.5 µl of 1X KAPA 2G Fast Ready Mix (Sigma-Aldrich, France), 0.5 µl of each barcode (Nextera Index Sequences in http://seq.liai.org/204-2/), 10.5 µl of ultrapure water and 1 µl of DNA for a final volume of 25 µl. PCR conditions were as follows: initial denaturation at 98 °C for 30 s followed by 8 cycles of 98 °C for 10 s, 60 °C for 20 s, 72 °C for 30 s and a final extension at 72 °C for 2 min. Again, each PCR product was run on 1% agarose gel at 100 V for 20 min in an electrophoresis chamber (Mupid®-One) to be sure that barcodes were well added. Negative controls (with "ultrapure" (UV treated) water) were performed for each of the steps described above. Incubation (37 °C for 30 min, 85 °C for 15 min) with USB ExoSAP-IT PCR Product Cleanup (Thermofisher, France) was then performed to degrade any free and unligated primers/barcodes/Illumina adapters. The concentration of all PCR products were normalized with a 96 well SequalPrep Normalization Plate (Thermofisher, France). Amplicons were pooled and concentrated by using the Wizard SV Gel and PCR Clean up Kit (Promega, France). Amplicons were sequenced using Illumina 2 × 300 bp MiSeq sequencing (FASTERIS SA, Switzerland).

### Processing of 16S sequences

The analysis of the raw sequences was done using the QIIME2 software and the standard pipeline of DADA2 [67–69]. Briefly, raw reads were demultiplexed, quality checked and trimmed to remove primer regions, paired ends were assembled, chimeric sequences were discarded, and reads were denoised. DADA2 infers a list of Amplicon Sequence Variants (ASVs). Sequences were aligned against the SILVA 138 reference database distributed by the Silva project [70, 71]. A phylogenetic tree was constructed using *q2-fragment-insertion* command from QIIME2, which uses the SEPP insertion tool with default parameters [72, 73]. Based on the classification, ASVs matching “Archaea”, “Eukaryota” and “Unassigned” were removed. ASVs represented by a single sequence in the ensemble of samples were also removed. A rarefaction analysis for each sample showed that two skin mucus samples had lower sampling depth than the others (*Boops boops*, 4188 reads and *Diplodus annularis*, 541 reads). These two samples were discarded and the data were rarefied to 15,100 sequences (the third lowest sampling depth). Finally, only skin or gill mucus samples with N > 3 within each fish species were used for downstream analysis (12 species represented in studying gill mucus microbiota and 11 species for skin mucus).

### Data and statistical analyses

#### Ecological traits

The ecology of each fish species within the sparid family was described using a set of 4 categorical traits describing position in water column, schooling behavior, diet and living environment (sandy, muddy, rocky or grassy). Values were taken from the FishBase (www.fishbase.org) database and the distribution of trait values among the sparid fish species is described in Additional file 1: Table S2.

#### Data analyses

Microbial alpha diversity was calculated using Shannon and Faith’s phylogenetic indices as implemented in the R package *phyloseq* [74]. As these data do not show normal distributions, we performed Kruskal–Wallis rank sum tests (KW) and post hoc Conover-Iman (CI) tests for multiple comparisons with Benjamini–Hochberg correction, to detect significant differences in diversity indices between fish species, habitat (skin and gill mucus and planktonic communities) and ecological fish traits. Principal coordinates analysis (PCoA) using both Bray–Curtis, based on ASVs’ abundance, and weighted Unifrac distance, which takes into account both the ASVs’ abundance and their phylogenetic relationships, was used to assess the differences between the microbiota of the different fish species. Permutational multivariate analysis of variance (PERMANOVA, as implemented in the *adonis* function of the R package *vegan*) and pairwise comparisons for weighted Unifrac and Bray–Curtis indices (1000 permutations) were used to evaluate statistically significant differences of PCoA groups between fish species, habitat, ecological fish traits, as well as the effect of environmental variables measured by the SOMLIT (Service d’Observation en Milieu Littoral, https://www.somlit.fr/) on each sampling date (temperature, salinity, oxygen, NH_4_, NO_3_, NO_2_, PO_4_ and SiOH_4_, Additional file 1: Table S3). The number of shared ASVs among skin mucus, gill mucus microbiota and planktonic communities was calculated and represented using a Venn diagram (using the rarefied ASVs table). To assess how each microbial taxon contributed to the dissimilarity between skin mucus, gill mucus and water bacterial communities, we performed a Linear discriminant analysis Effect Size (LEfSe) [75]. LEfSe provides Linear Discriminant Analysis (LDA) scores for the bacteria taxa contributing the most to the differences between bacterial communities. We calculated relative abundances (i.e., total sum scaling after rarefaction) of microbial taxa (phyla, class, family and genus) showing a significant contribution to the dissimilarity between skin mucus, gill mucus and water bacterial communities.

In order to test whether interspecific dissimilarities in skin or gill microbiota could be explained by the host phylogeny (phylosymbiosis), we first constructed the phylogenetic tree of sparids by using *Dicentrarchus labrax* as outgroup [76]. Phylogenetic analysis was performed by concatenating 4 sparid gene sequences for each fish species: the mitochondrial 16S rRNA, rhodopsin, cytochrome b and cytochrome c oxidase subunit 1 available on the National Center of Biotechnology Information database. Sequences of each coding gene were aligned using codon positions in MEGA X [77]. The nucleotide substitution model for the host mitochondrial 16S rRNA gene was estimated via Akaike Information Criterion using jModelTest v.2.0 [78]. The tree was constructed from the concatenated dataset with IQ-TREE program [79] using appropriate codon models for rhodopsin, cytochrome b and cytochrome c oxidase 1. The partition (rhodopsin: CODON, cytochrome b: CODON2, cytochrome oxidase: CODON2, 16S: GTR + G) was applied and maximum likelihood analysis were run with 10,000 ultrafast bootstrap replicates. A matrix of patristic distances was then generated with the *vegan* and *ape* R packages [80, 81]. The correlation between interspecific dissimilarities in skin or gill microbiota and host phylogeny was then assessed using Mantel tests based on Pearson’s coefficient, using *vegan* with 1000 permutations. Two methods were used to generate the dissimilarity matrix for skin and gill mucus microbiota: the first method is based on the random selection of one individual per fish species and the second matrix is generated by averaging the microbial taxa among individuals of each fish species, before performing a Mantel test for both methods [23, 25, 26]. The external mucus is in intimate contact with the aquatic environment and there is probably a continuous exchange of bacteria between these two compartments. Some of the bacteria present in the external mucus could be considered as "contaminations" from the surrounding water and be only transient. These phylosymbiosis analyses were therefore performed on the “entire” gills’ microbiota (considering sequences present in water samples) but also on the tissue-specific gills’ microbiota (without sequences from water) in order to avoid as much as possible the putative influence of these transient bacteria. The same analyses were performed based on the core microbiota of each host species, which is the most stable part of their microbiota. To determine skin and gill mucus core microbiota, we identified ASVs that were present in 90% of individuals for each host species.

Correlations between *Lamellodiscus* diversity (i.e. species richness, the number of different *Lamellodiscus* species in each fish individual) and gill microbiota diversity (Faith’s and Shannon index) were computed and their significance assessed using Pearson’s correlation tests. Finally, Mantel tests were performed to analyze associations between the composition and total abundance of all *Lamellodiscus* species and gill mucus microbial communities. We also tested the influence of each *Lamellodiscus* species abundance on the composition of the gill mucus microbiota, by performing one Mantel test per *Lamellodiscus* species. Spearman’s rank correlation was used to investigate the putative link between the abundance of *Lamellodiscus* species and the composition of gill microbial communities at the genus level. Again, these analyses were performed with and without considering sequences from water samples. A correlation between the abundance of a *Lamellodiscus* species and the abundance of a bacterial genera was considered to be significant when *p*-*value* < 0.05, when the bacterial genus is represented at least by 75 sequences (representing 0.1% of total sequences after rarefaction, after removing water sequences) and in 3 fish gill mucus samples.

## Results

A total of 4,851,954 sequences assigned to bacteria (i.e. filtering out reads belonging to Archaea, Eukaryota and unassigned reads) were obtained across all samples. After rarefaction analysis and by taking into account only samples with N > 3 for each category, a total of 10,610 ASVs were recovered from the gill mucus, skin mucus and surrounding water samples (58, 48 and 10 samples respectively). The most abundant bacterial group was *Proteobacteria* (68% of all sequences), followed by *Firmicutes* (14%), *Bacteroidetes* (5%) and *Actinobacteria* (3%).

### Differences in the diversity of microbiota

**Fig. 1:**
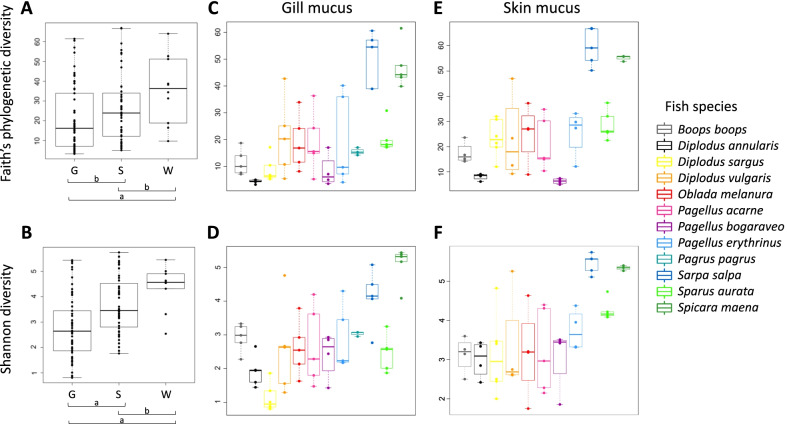
Comparison of alpha diversity values between habitats and host fish species. Faith’s phylogenetic diversity and Shannon diversity index for skin mucus (S), gill mucus (G) and water communities (W) (**A**, **B**) and within host fish species for gill mucus (**C**, **D**) and skin mucus (**E**, **F**). Significant (a) and non-significant (b) differences between tissue are indicated (Conover-Iman test (post hoc test), *p *< alpha/2 = 0.025). Significant and non-significant differences in skin and gill mucus diversity between fish species are available in Additional file 1: Table S4

**Fig. 2:**
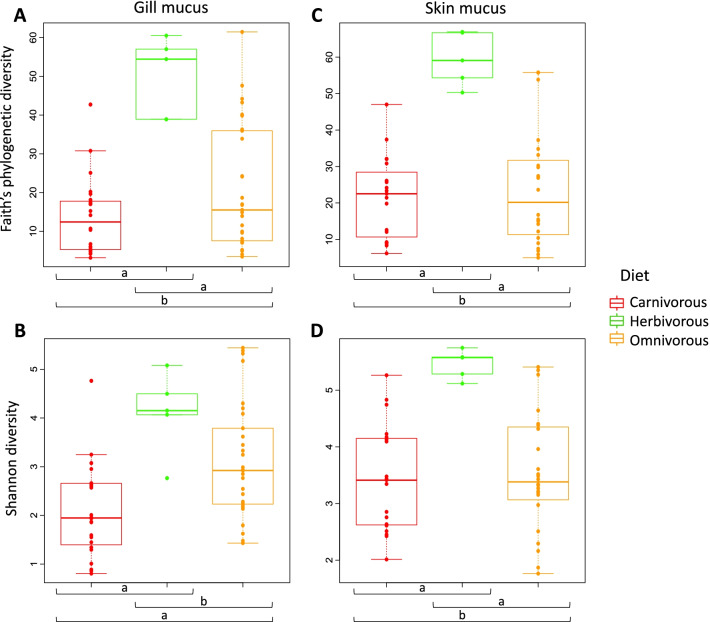
Comparison of alpha diversity values between host fish diet. Faith’s phylogenetic diversity and Shannon diversity index for gill mucus (**A**, **B**) and skin mucus (**C**, **D**) for each diet category. Significant (a) and non-significant (b) differences between diet groups are indicated (Conover-Iman test (post hoc test), *p* < alpha/2 = 0.025)

We measured the diversity within communities (alpha diversity), using two metrics: the Shannon diversity index reflecting taxonomic richness and evenness, and the Faith’s phylogenetic index that reflects the phylogenetic richness. When comparing diversity metrics, we found significant differences between the bacterial community of the skin mucus, gill mucus and the surrounding water (KW test, *p* < 0.05). For both alpha diversity metrics, there was no significant differences in diversity between water and skin mucus (KW test, *p* < 0.05; CI tests, *p* > alpha/2 = 0.025, Fig. 1A, B). However, the Shannon diversity of both skin mucus and water communities was significantly higher than for gill mucus (KW test, *p* < 0.05; CI tests, *p* < alpha/2 = 0.025, Fig. 1A, B) showing that these two habitats harbor much more diverse bacterial communities than gill mucus. For the Faith’s phylogenetic index, only water and gill mucus communities were significantly different from each other (KW test, *p* < 0.05; CI tests, *p* < alpha/2 = 0.025; skin mucus vs gill mucus, *p* = 0.029). Moreover, we found significant differences in diversity when comparing host fish species (KW test, *p* < 0.001 for both metrics), which can be probably explained by the highest bacterial diversity in skin and gill mucus of *Spicara maena* and *Sarpa salpa* compared to the other fish species (KW test, *p* < 0.001; CI test *p* < alpha/2 = 0.025 Fig. 1C–F; Additional file 1: Table S4). Finally, diet showed also a significant effect on both skin and gill mucus diversity (KW test, *p* < 0.05 for both metrics), with in most cases a higher diversity for herbivorous fish species compared to carnivorous and omnivorous species (KW test, *p* < 0.05; CI tests, *p* < alpha/2 = 0.025, Fig. 2), where these last two harbor generally a similar microbial diversity (KW test, *p* < 0.05; CI tests, *p* > alpha/2 = 0.025) (Fig. 2).

### High dissimilarities among fish microbiota composition and phylosymbiosis

To determine which factors explain the variability between and within skin mucus, gill mucus and water microbiota, we used two metrics: the Bray–Curtis dissimilarity index (BC), which takes into account the relative abundances of each ASV, and the weighted Unifrac distance (WU), which incorporates both the relative abundances of each ASV and phylogenetic relationships between ASVs. Principal coordinate analysis (PCoA) was used to plot both BC and WU distances.

**Fig. 3:**
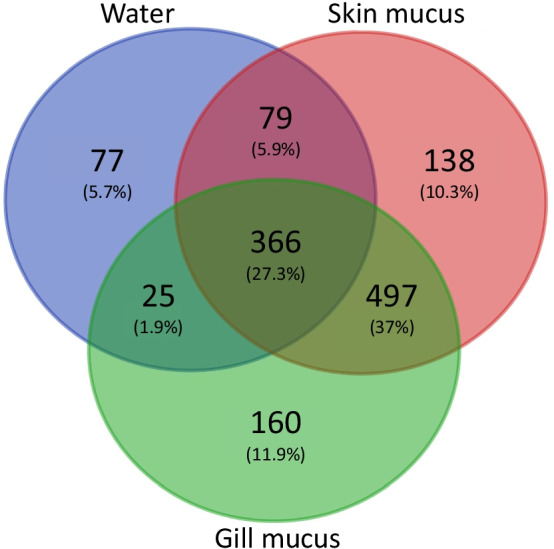
Venn diagram representing shared ASVs between skin mucus (red), gill mucus (green) and water (blue). Based on 0.005% abundance cutoff

Our first results showed significant differences between bacterial communities from skin mucus, gill mucus and surrounding water (PERMANOVA for BC and WU respectively: *p* < 0.001 (R^2^ = 0.087); *p* < 0.001 (R^2^ = 0.093)) (Additional file 1: Fig. S1). Both BC and WU showed smaller significant differences between skin mucus and gill mucus communities (*p* < 0.01; F = 2.45 and F = 2.33 respectively) than between skin mucus and water (*p* < 0.01; F = 4.48 and F = 5.14 respectively) or between gill mucus and water communities (*p* < 0.01; F = 4.42 and F = 3.25 respectively). Skin mucus and gill mucus shared 27.3% of ASVs with water and 37% between them (Fig. 3).

**Fig. 4:**
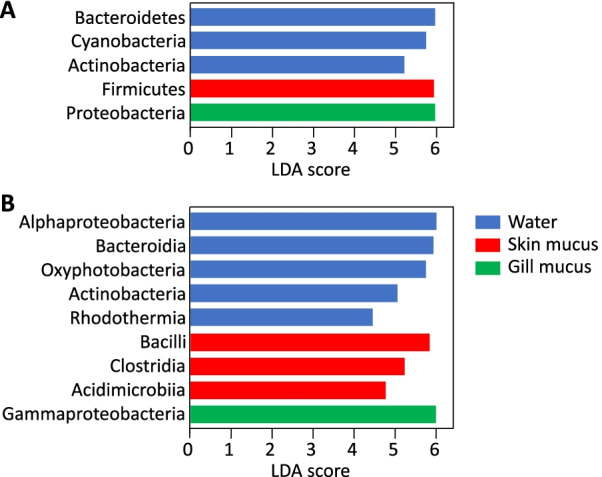
Most contributing taxa to differences between water (blue), skin (red) and gill (green) bacterial communities. **A** phylum, **B** class level. LDA scores were calculated using Linear discriminant analysis Effect Size (LEfSe), only bacterial taxa that raised an LDA score > 4 are shown

Compared to fish skin mucus or water communities, fish gill mucus microbiota contained significantly higher abundances of sequences of *Proteobacteria* (71 ± 25% in relative abundance (i.e., total sum scaling method, after rarefaction)) and *Gammaproteobacteria* (64 ± 28%), especially in *Vibrionaceae* (24 ± 32%) and *Pseudomonadaceae* (12 ± 22%) (LEfSe analysis, Fig. 4, Additional file 1: Fig. S2). The two most abundant genera were *Photobacterium* (13 ± 24%) and *Pseudomonas* (12 ± 22%) (confirmed by the LEfSe analysis; Additional file 1: Fig. S2) compared to skin mucus microbiota that harbored more *Firmicutes* (19 ± 21%), *Bacili* (11 ± 21%), *Clostridia* (3 ± 8%), *Rhodobacteraceae* (8 ± 9%) and *Moraxellaceae* (13 ± 18%). The most abundant genera in skin mucus microbiota were *Psychrobacter* (12 ± 18%) and *Exiguobacterium* (3 ± 9%). Sea water communities were especially rich in *Bacteroidetes* (20 ± 11%), *Cyanobacteria* (11 ± 6%) and classes *Alphaproteobacteria* (31 ± 16%) and *Bacteroidia* (19 ± 11%). Families significantly more abundant within the water compared to fish microbiota were *Flavobacteriales* (18 ± 10%) and *Synechococcales* (10 ± 6%) and *Synechococcus* was the most abundant genus (10 ± 6%) (LEfSe analysis: Fig. 4, Additional file 1: Fig. S2). Despite their constant exposition to the same water microbial communities, skin and gill mucus harbor different microbial abundances both from each other and from the surrounding environment.

**Fig. 5:**
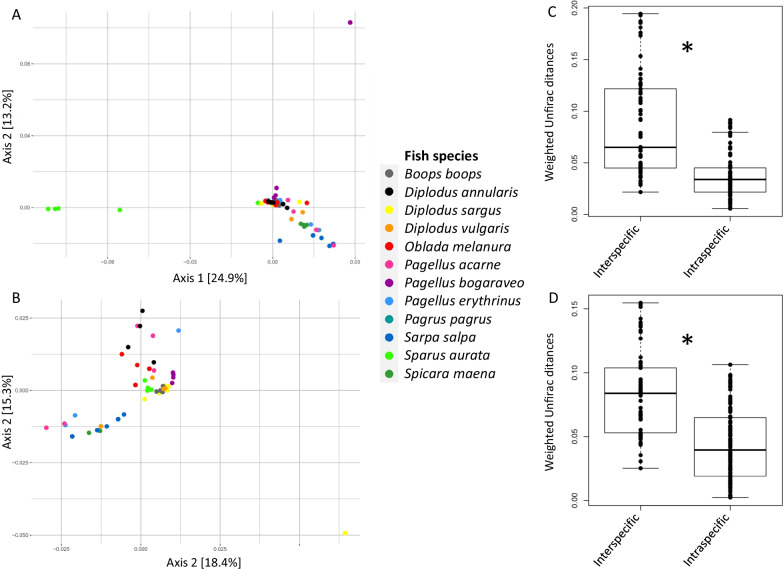
Dissimilarities between bacterial communities based on weighted Unifrac dissimilarity values. PCoA plot representing all fish gill mucus (**A**) and skin mucus (**B**) microbiota included in this study. Color indicates fish species. Intraspecific and interspecific values based on weighted Unifrac dissimilarity within gill mucus (**C**) and skin mucus (**D**) microbiota. *Significant differences between intraspecific and interspecific values (Kruskal–Wallis test, *p* < 0.05)

Interspecific dissimilarities among fish skin mucus or gill mucus microbiota were significantly higher than intraspecific dissimilarities (KW test (non-normal data distribution) performed on BC and WU dissimilarity values for both skin and gill mucus microbiota, *p* < 0.001) (Fig. 5C, D). For example, interspecific WU dissimilarities were 1.9 times and 2.3 times higher than intraspecific ones for gill mucus (0.08 ± 0.03 vs 0.04 ± 0.03) and skin mucus (0.09 ± 0.05 vs 0.04 ± 0.02) microbiota respectively (Fig. 5C, D) (around 1.3 times for BC dissimilarity values, Additional file 1: Fig. S3C, D). Moreover, a significant host fish species effect on the variability of skin and gill mucus microbial communities was found (PERMANOVA, *p* < 0.001, 0.41 < R^2^ < 0.52 which means that individuals within a given fish species harbor significantly more similar bacterial communities than with fish from other species (Fig. 5A, B; Additional file 1: Fig. S3A, B and Table 2). Most pairwise comparisons between fish species (for both skin and gill mucus microbiota) were significant (*p* < 0.05), with the exception of a few associations involving *Pagellus acarne*, *Pagellus erythrinus*, *Diplodus vulgaris* or *Pagrus pagrus* (Additional file 1: Table S5), which is coherent with the high dissimilarity values among individuals within these fish species (Additional file 1: Fig. S4). In order to understand which other factors determine external microbiota composition, additional PERMANOVAs were also performed with fish ecological traits. Interspecific differences in both skin and gill microbiota were not predicted by schooling behavior of the host (Table 2), whereas diet had an effect on both skin and gill microbiota (WU dissimilarity values, *p* < 0.001, R^2^ = 0.12) (Table 2). The other ecological traits seemed to have also a weak, but significant, effect on fish microbiota depending on metrics and habitats considered (0.04 < R^2^ < 0.07, Table 2). For example, based on WU distances, both rocky and grassy environments and position in the water column had no significant effect on fish gill microbiota, whereas these samples clustered further apart based on BC dissimilarities. These results suggest that the differences in skin or gill communities involve closely related microbial taxa. Finally, the skin and gill mucus microbiota structure was also influenced by temperature (*p* < 0.05, WU, R^2^ = 0.11 and R^2^ = 0.05 respectively), and salinity was linked to the skin microbiota structure (*p* < 0.05, WU, R^2^ = 0.05).

**Table 2:** Results of PERMANOVAs on host and ecological factors explaining the variability in bacterial communities’ composition

	Gill mucus				Skin mucus			
	Bray–Curtis		W-Unifrac		Bray–Curtis		W-Unifrac	
	*p* value	R^2^	*p* value	R^2^	*p* value	R^2^	*p* value	R^2^
Fish species	**< 0.001**	0.470	**< 0.001**	0.470	**< 0.001**	0.524	**< 0.001**	0.406
Position in water column^ET^	**< 0.001**	0.045	0.225	–	**0.009**	0.041	0.277	–
Schooling behavior^ET^	0.090	–	0.874	–	–	–	–	–
Diet^ET^	**< 0.001**	0.097	**< 0.001**	0.125	**< 0.001**	0.138	**< 0.001**	0.124
Sandy environment^ET^	**0.001**	0.043	**0.033**	0.032	**0.014**	0.039	0.139	–
Muddy environment^ET^	**< 0.001**	0.050	**< 0.001**	0.053	**< 0.001**	0.065	**< 0.001**	0.058
Rocky environment^ET^	**0.001**	0.044	0.081	–	**0.001**	0.052	**0.001**	0.060
Grassy environment^ET^	**0.003**	0.036	0.150	–	**< 0.001**	0.056	**0.003**	0.048

ET, ecological trait

Significant *p* values are in bold (PERMANOVA, *p* < 0.05)

We computed correlations between interspecific differences in skin or gill microbiota and phylogenetic distances between host fish species (Additional file 1: Fig. S5) using two methods. The first consisted of randomly selecting one individual per fish species, and the second was based on the average of microbial taxa across individuals of each fish species, before performing a Mantel test. Both methods did not detect any significant correlation between microbial distances and host phylogeny by considering either skin or gill microbiota (BC or WU distances, *p* > 0.1), with or without considering sequences present in water samples (Additional file 1: Table S6). No phylosymbiosis signal was found considering skin or gill mucus core microbiota (Additional file 1: Table S6).

### *Lamellodiscus* composition and abundance and their link with microbial taxa

A total of 21 *Lamellodiscus* species (including *Furnestinia echeneis*, as this species is considered as a *Lamellodiscus* (see [58])) were found in the gills across all fish species (Additional file 1: Table S7). The pattern of presence/absence of *Lamellodiscus* species observed here within fish species is in accordance with previous studies [48–58]. Among the *Lamellodiscus* species characterized, there are specific species (with one or two host species), such as *Furnestinia echeneis* (on *Sparus aurata*) or *Lamellodiscus parisi* (*Sarpa salpa*), and generalist species (that parasitized more than 2 host species) such as *Lamellodiscus elegans*, *Lamellodiscus ergensi* or *Lamellodiscus ignoratus. Boops boops* was the only fish species with no *Lamellodiscus* individuals found*.* Abundances and species richness of *Lamellodiscus* within each individual fish are summarized in Additional file 1: Table S7.

**Fig. 6:**
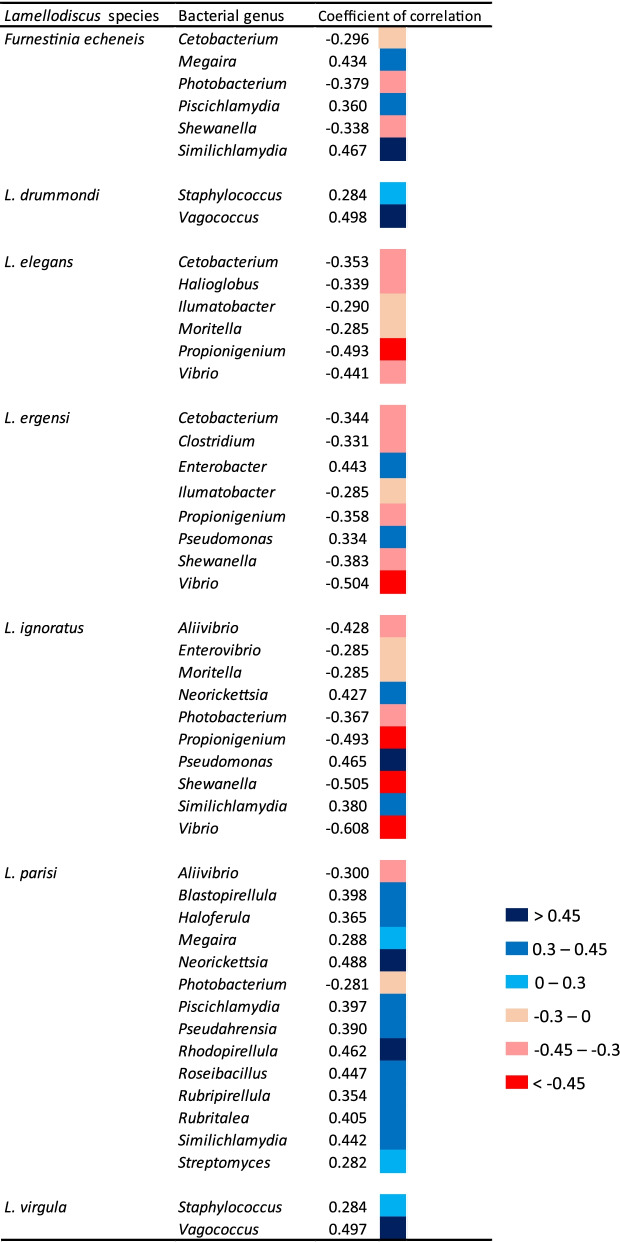
Significant Spearman correlation coefficients between the abundances of *Lamellodiscus* and bacterial genus in fish gills. The color of each cell represents positive (blue) and negative (red) correlations coefficients

Both Faith’s phylogenetic index and Shannon diversity in gill mucus microbiota were negatively correlated with *Lamellodiscus* diversity (i.e. species richness) (Pearson correlation test, *p* < 0.05, R = − 0.27 and R = − 0.50 for Faith’s phylogenetic and Shannon index respectively), which means that an increase of parasite diversity is linked to a decrease of gill microbiota diversity. Moreover, the composition and abundance of *Lamellodiscus* species were significantly correlated with the bacterial composition of fish gill microbiota (Mantel test, *p* < 0.001, R = 0.42). More specifically, 8 *Lamellodiscus* species structure the variability of bacterial communities: *Furnestinia echeneis* (*p* < 0.001, R = 0.14), *Lamellodiscus drummondi* (*p* < 0.01, R = 0.13), *Lamellodiscus elegans* (*p* < 0.01, R = 0.12), *Lamellodiscus ergensi* (*p* < 0.001, R = 0.18), *Lamellodiscus ignoratus* (*p* < 0.001, R = 0.19), *Lamellodiscus mirandus* (*p* < 0.01, R = 0.10), *Lamellodiscus parisi* (*p* < 0.01, R = 0.12) and *Lamellodiscus virgula* (*p* < 0.01, R = 0.12), (Fig. 6, Additional file 1: Table S8). To elucidate potential correlations between parasites and gill mucus microbiota, we quantified how the relative abundance of microbial taxa was related to parasite composition and abundance. Spearman correlation coefficient analyses indicated that the abundances of some *Lamellodiscus* species displayed significant positive or negative correlations with the relative abundance of given bacterial genera (Fig. 6). For example, the abundance of *Lamellodiscus drummondi* and *Lamellodiscus virgula* (parasites of *Pagellus acarne*) were positively correlated with the bacterial genera *Staphylococcus and Vagococcus* (R = 0.28 and R = 0.50 respectively) whereas the abundance of *Lamellodiscus parisi* (parasite of *Sarpa salpa*) was positively correlated with 12 bacterial genera, in particular with *Neorickettsia* (R = 0.49) and *Rhodopirellula* (R = 0.46). The abundance of the three generalist *Lamellodiscus* species *Lamellodiscus elegans*, *Lamellodiscus ergensi* and *Lamellodiscus ignoratus* were correlated with *Halioglobus* (R = − 0.34), *Clostridum* (R = − 0.33) and *Enterovibrio* (R = − 0.29) respectively, but their abundance is also negatively correlated with *Vibrio* and *Propionigenium*. The bacterial genus *Moritella* was negatively correlated with the abundance of *Lamellodiscus elegans and Lamellodiscus ignoratus*, whereas the abundance of *Lamellodiscus ergensi* and *Lamellodiscus ignoratus* were positively and negatively correlated with *Pseudomonas* and *Shewanella* respectively. The abundance of *Lamellodiscus parisi* and *Furnestinia echeneis* were also negatively correlated with the abundance of *Photobacterium* (Fig. 6). By considering the entire gill microbiota (with sequences from the surrounding water), only the abundance of *Lamellodiscus elegans* (*p* < 0.001, R = 0.17), *Lamellodiscus ergensi* (*p* < 0.001, R = 0.19) and *Lamellodiscus ignoratus* (*p* < 0.001, R = 0.17) displayed a significant link with bacterial communities. Spearman correlation analyses identified more significant correlations between the abundance of these three *Lamellodiscus* and bacterial genera (Additional file 1: Fig. S6). Most significant correlations identified in the first place (without water sequences) were also found in this second analysis, which reinforced the significant link between *Lamellodiscus* species and bacterial genera. Finally, a certain number of bacterial taxa appeared to be particularly associated to *Boops boops*, the only fish species in this study that is not parasitized by *Lamellodiscus* species. We identified that gill mucus microbiota of *Boops boops* contained significantly higher abundances of *Fusobacteria* (12.1%; relative abundance), while this phylum is nearly absent in other fish species (average of 0.9% among all other fish species) (LEfSe analysis, Additional file 1: Fig. S7). One *Fusobacteria* and two *Proteobacteria* families are also significantly more abundant in *Boops boops*: *Fusobacteriaceae* (12.1% vs 0.9%), *Shewanellaceae* (10.1% vs 1.6%) and *Vibrionaceae* (71.7% vs 18.8%). Finally, we also identified that three genera (one in each bacterial family mentioned in the previous sentence) were particularly abundant in *Boops boops*: *Cetobacterium* (11.4% vs 0.8%), *Shewenella* (10.1% vs 1.6%) and *Vibrio* (54% vs 3.9%) (LEfSe analysis, Additional file 1: Fig. S7).

## Discussion

### Fish skin and gill mucus harbor different microbial diversity and composition

Each habitat (skin, gills or water) harbors different microbial abundances both in terms of diversity and composition. Consistent with previous studies, skin and gill mucus microbiota are largely dominated by *Proteobacteria*, and other less abundant phyla such as *Firmicutes* or *Fusobacteria* [23, 64, 82–84]. Despite these common phyla, skin and gill microbiota harbor different abundances both from each other and from the surrounding water, while they are continuously exposed to the microbiota of the aquatic environment; this is also true at lower taxonomic levels [84]. A high number of shared microbial taxa were found between skin mucus, gill mucus and water communities (27.3% of shared taxa). Several studies on fish external microbiota (on both farmed and natural fish populations) have already reported a large number of common microbial taxa between gill mucus, skin mucus, and water sample [64, 85, 86], which could be explain by the important role of surrounding seawater as a source of bacteria for the external mucus [87]. However, as these two compartments are constantly in contact, some bacteria may also appear only transiently in the external mucus microbiota (and can be considered as not truly shared microbial taxa).

Skin mucus contained significantly higher abundances of *Firmicutes* and *Bacilli*, while gill mucus harbored more *Proteobacteria* and *Gammaproteobacteria* and water communities contained more *Bacteroidetes*, *Cyanobacteria* and *Actinobacteria*. Regarding diversity, gill mucus harbored a lower alpha diversity, compared to skin mucus and water bacterial communities. Other freshwater or seawater fish species display a similar trend, with a lower diversity in gill mucus compared to the skin mucus [15, 64, 88]. Moreover, we found that sparid skin and gill mucus are also highly species-specific, both in terms of diversity and composition, as previously reported in several other fish families such as Chaetodontidae, Serranidae, Labridae or Mullidae [23, 24, 64, 82, 89]. The external mucus (skin and gills) is the main surface of exchange between fish and their surrounding environment. It represents an important protective barrier against pathogens, since it reduces the colonization by pathogenic organisms (bacteria, virus or eukaryotic organisms) [9, 30, 90]. The differences in terms of bacterial diversity and composition between skin and gill mucus could also be due to the production of tissue-specific metabolites, the immune system or functions of these two habitats [40, 91, 92]. For example, the interactions between microbial communities and the immune system can differ between skin and gills [9, 93]. Gills, in addition to their protective role, possess unique functions such as gas exchange (gills being the most important respiratory organ in fish) or waste excretion. Ammonia excretion in fish occurs particularly in gill mucus and could influence the colonization by microbial communities [94]. Several studies also showed that some pathogenic bacteria were differentially attracted by skin mucus or gill mucus [95, 96]. All these observations suggest that fish skin and gill mucus microbiota diversity and composition are not simple reflections of the microbial assemblages of their habitat, but likely result from selective mechanisms that differ according to each habitat (skin or gills) and fish species.

### Dissimilarities among microbiota are not explained by phylosymbiosis

We found that the variability of skin and gill mucus microbiota was more similar between individuals from the same species than between individuals from different species, supporting a species-specificity of fish microbiota as previously reported [23, 24, 82, 89]. However, these interspecific dissimilarities between fish species for the skin and gill mucus microbiota are not correlated with the host phylogeny. This absence of phylosymbiotic pattern was inferred using both methods (random sampling or averaging of fish microbiota), with and without considering bacterial sequences present in water samples. Phylosymbiosis was mostly reported in studies investigating gut microbiota of terrestrial animals, including hominids [97], mammals [26], birds [98] and insects [25]. In natural populations, identifying a relationship between skin or gill microbiota of fish and the host phylogeny is equivocal, as both environmental factors and the influence of host traits can covary and contribute to microbial assemblages. Despite this putative environmental influence, two studies inferred phylosymbiosis in mammals (Artiodactyla and Perissodactyla [99]) and in the skin microbiota of coral reef fish [23]. The absence of a significant phylogenetic signal in our study could be explained by several different factors such as the effects of the surrounding environment, fish ecological traits or fish status. Several studies already suggested that seasonal variations and environmental factors influence fish microbiota composition. These studies investigated the influence of environmental factors on fish gut microbiota, and highlighted that temperature or salinity can be highly variable and can heavily influence the composition of gut bacterial communities [20, 100–107]. Large or even complete shifts in bacterial abundances have been reported after acclimation to salinity or between seasons [20, 103, 105, 106, 108]. Recently, seasonal changes and associated fluctuations in environmental factors (such as salinity [17, 108, 109], pH [110], geographic locations [107], seasonality or temperatures [18–20, 111, 112]) were reported to affect the external microbial structure: the external mucus microbiota of aquatic vertebrates was found to be highly variable and dynamic in response to environmental conditions. Moreover, water microbial communities are also in fluctuating habitats, known to be strongly marked by seasonality and rhythmicity of environmental factors such as temperature and salinity [21, 111, 112]. Due to upwellings or downwellings, eutrophication or phytoplankton blooms, bacterial communities in the water column change, thus modifying exposure to teleost fish and the interaction between fish host and its microbiota. In this study, we sampled fish individuals over a two-year period during different seasons. To collect the 15 species of wild sparids used in this study, long-term sampling was required (over a two-year period). The significant intraspecific and interspecific dissimilarities we found within each fish species and between species may therefore be due to environmental variations between seasons, which may interfer with a phylosymbiosis pattern.

We also tested whether interspecific dissimilarities observed among fish skin and gill mucus could be predicted by fish ecological traits. Diet is the most important ecological trait in this study linked to both skin and gill microbiota diversity and structure (R^2^ = 0.12 in WU for both skin and gill mucus microbial communities). Recently, Escalas and coll. [113] highlighted that the dissimilarities between gut microbiota in sparids (study based on 12 species) were not explained by their phylogeny but by diet which appeared to be the most significant factor that directly affects the diversity and composition of fish gut microbiota [113–116]. Gut bacterial diversity is generally lower in carnivores, and increases in omnivores and herbivores [117], a trend also observed in the present study. Recent studies showed a significant effect of diet also on the fish skin and gill microbiota [23, 89, 99]. Several hypotheses can be proposed to explain this diet effect. The first hypothesis is a shift in fish gut microbiota that could be indirectly transferred to the skin through the aquatic environment from fish feces [23]. Alternatively, variations in diet are known to result in changes within the external mucus in terms of secretion and production of metabolites which subsequently can affect microbial diversity and composition [40, 41, 118]. Therefore, diet could be a stronger determinant of the whole microbial community structure in teleost fish species but the characterization of such metabolites and their correlation with diet has yet to be confirmed. Fish gut microbiota has been reported to be strongly linked to fish immunity [119, 120]. A change in diet may then act on fish gut microbiota, which may influence fish immune performance and immune gene expression and possibly affect skin microbiota, by increasing or decreasing the secretion of antimicrobial metabolites for example.

Teleost fish species are covered by mucus, where chemical composition and thickness can be highly variable [40, 118, 121] depending on the fish immune system [9]. Indeed, differences in skin or gill mucus immunology were observed and related to stressful conditions such as starvation [122], fish health status (presence of parasites or pathogenic bacteria, [40, 123, 124], age [125] or host genotype [126]). Therefore, all these different factors shaping individual fish immune system could explain the high level of intraspecific variability, especially the infection status of each fish individual observed in this study (considering only *Lamellodiscus* species, some hosts do not harbor any parasites whereas others are highly parasitized) and could explain the absence of phylosymbiosis. It is also possible that the host immune system is a better regulator of host-microbiota interactions than ecological traits or phylogeny. Several studies on other animals showed that the expression of host immune genes can explain the variations in microbial community structure and can be essential for the establishment of host-specific microbiota [127–129].

### A strong link between microbial taxa and parasite diversity and abundance

In this study, we examined the associations between the composition and abundance of different ectoparasitic monogenean species and gill microbiota composition in a wild Mediterranean teleost fish family, the Sparidae. We found that the abundances of 8 monogenean species (*Funrestina echeneis*, *Lamellodiscus drummondi, Lamellodiscus elegans*, *Lamellodiscus ergensi*, *Lamellodiscus ignoratus*, *Lamellodiscus mirandus*, *Lamellodiscus parisi* and *Lamellodiscus virgula*) were positively or negatively linked to the abundance of some microbial genera. To our knowledge, this is the first study showing that the bacterial abundance in gill mucus of teleost fish varies according to the abundance of ectoparasitic species that colonize the host. One part of these significant associations seemed to be species-specific, i.e. a given *Lamellodiscus* species is associated with a unique microbial taxa. Moreover, the increase or decrease in abundance of given microbial taxa is linked in a same way to abundances of several *Lamellodiscus* species (both negative and positive correlations). We found that abundance of *Vibrio* was negatively correlated with the abundance of three parasitic species, *Lamellodiscus ignoratus*, *Lamellodiscus ergensi* and *Lamellodiscus elegans*, and that *Photobacterium* was associated with the parasites* Furnestinia echeneis, Lamellodiscus ignoratus* and *Lamellodiscus parisi.* Negative correlations between the abundance of these two potential bacterial pathogens, *Vibrio* and *Photobacterium*, and a high number of intestinal endoparasites (28 parasitic species: digeneans, monogeneans, nematodes, cestodes…) have been reported in three tropical fish species, *Epinephelus fuscoguttatus*, *Epinephelus sexfasciatus* and *Atule mate* [45]. In this study of Hennersdorf and coll. [45], the microbiota was suggested to interact with parasites in many ways, affecting their abilities (by inhibiting or enhancing) to colonize fish gills, an observation already reported in previous papers where a relation between gut microbiota and intestinal parasites in animals was established. For example, in mice, a well-known parasitic nematode, *Trichuris muris*, requires bacterial interactions to establish infections, whereas the bacterial taxa *Lactobacillus casei* and *Bifidobacterium animalis* reduce the abundance of the nematode *Trichinella spiralis* [130, 131].

Commensal microbiota in external mucosal surfaces plays an important role in fish homeostasis [132] in avoiding the proliferation of pathogens. Recent studies highlighted that some microorganisms synthetize molecules against fish pathogens (antibacterial and antifungal molecules) [14, 15]. Interestingly, we found that *Fusobacteria* and the three genera *Shewanella*, *Cetobacterium* and *Vibrio* were significantly enriched in the only fish species not parasitized by *Lamellodiscus* in this study, *Boops boops*. A similar result was previously reported by Reverter and coll. in 2020 [41] who found among others, higher abundances of *Fusobacteria*, *Spirochaetaceae*, *Shewanellaceae* and *Vibrionaceae* (*Vibrio* sp.) in the gill mucus of the unparasitized butterflyfish species*, Chaetodon lunalatus,* compared to other *Chaetodon* species. Similar hypotheses and conclusions can therefore be drawn here. Briefly, anaerobic bacteria *Fusobacteria* are known for their beneficial effects on mammals: they strengthen the external protective barrier against pathogens by increasing mucus production and have anti-inflammatory effects [133, 134]. The authors hypothesized that the presence of *Fusobacteria* causes an increase in the thickness of the mucus layer and subsequently an increase of oxygen diffusion distance and a potential hypoxia [135]. They also found a positive correlation between *Fusobacteria* and three hemoglobin-derived peptides, which could play a role as antimicrobial and antiparasitic. Moreover, *Cetobacterium* (*Fusobacteria*) is also known to synthesize cobalamin, or vitamin B12, which prevents the growth of pathogens [84, 136, 137]. Altogether, these observations indicate that some bacteria within *Fusobacteria* and *Proteobacteria* (*Spirochaetaceae*, *Shewanellaceae* and *Vibrionaceae*) might play a role in preventing monogenean infection in *Boops boops*. Moreover, several studies have reported that some molecules present in fish mucus, potentially produced by microorganisms, induced the attachment of ectoparasites, such as copepods or monogeneans [38, 138]. For example, Ohashi and coll. [38] have shown that a glycoprotein present in skin mucus induces the attachment of *Neobenedenia girellia*, a monogenean ectoparasite of *Takifugu rubripes.* Finally, the interaction between microbiota and parasites could be also driven by changes induced by parasites. Antimicrobial molecules have been identified in numerous plathelminths parasitizing terrestrial gut animals: in nematodes such as *Ascaris suum* and *Strongyloides venezuelensis* [139, 140], and digenean species such as *Schistosoma* [141]. Therefore, molecules secreted by parasitic helminths may directly interact with the microbiota and create a favorable environment for their survival, and potentially increase the risk of other parasitic infections [142]. Studying these three-way interactions between pathogens, microbiota and the host is complex because many different beneficial or deleterious mechanisms can occur within and between these 3 compartments. Almost all previous studies focused on the impact of parasites on the microbiota and the physiology of their host [42–47]. However, the study of the parasite-associated microbiota, its influence on the parasite fitness, and its interactions with the abiotic and biotic environment and with its host (physiological traits and host microbiota), represent an emerging field of research that must also be developed in the next few years to better understand these tripartite associations [143, 144]. Further investigation is needed to determine in detail how these interactions take place and the impact, especially in terms of metabolites, that parasites and microbiota have on each other.

## Conclusion

Our study is among the very first to explore the diversity and structure of external mucus microbiota (both skin and gills) from a wild fish family. Fish skin and gill mucus harbor tissue-specific communities of bacteria despite persistent exposure to the surrounding water. We highlighted that both skin and gill mucus microbiota seem mainly shaped by host factors, host species and diet. We also reported the absence of a phylosymbiosis pattern for both skin and gill mucus microbiota within sparids, which may be explained by the influence of environmental or other host factors (ecological traits or fish heath status). In addition, we presented novel evidence about the links between gill mucus microbiota and ectoparasitic monogenean species in diversity and abundance. We pointed out that the abundances of some *Lamellodiscus* species were negatively or positively correlated with some microbial taxa. Several mechanisms, such as the protective and attractive roles of microorganisms, or the effect of parasites on fish microbiota, could act on the pattern of monogenean host specificity.

## Supplementary Information

**Additional file 1.** Table S1 to Table S8 and Figure S1 to Figure S7.

## Data Availability

Sequence data will be available upon publication in the NCBI Sequence Read Archive (SRA, https://www.ncbi.nlm.nih.gov/sra) database under the BioProject PRJNA748412.

## Abbreviations

ASV: Amplicon sequence variant
PERMANOVA: Permutational multivariate analysis of variance
PCoA: Principal coordinates analysis
LEfSe: Linear discriminant analysis Effect Size
BC: Bray–Curtis dissimilarity index
WU: Weighted Unifrac distance
KW: Kruskal–Wallis
CI: Conover-Iman
