# Supplementary material for: Diversity and structure of sparids external microbiota (Teleostei) and its link with monogenean ectoparasites
Source: Anim Microbiome. 2022 Apr 13;4:27. doi: 10.1186/s42523-022-00180-1 (PMC9009028; doi:10.1186/s42523-022-00180-1)
Supplement: Supplementary file 1 — Additional file 1. Table S1 to Table S8 and Figure S1 to Figure S7. [file 42523_2022_180_MOESM1_ESM.pdf]

Diversity and structure of sparids external microbiota (Teleostei) and its link with monogenean ectoparasites

Mathilde Scheifler[1*], Sophie Sanchez-Brosseau[1], Elodie Magnanou[1], Yves Desdevises[1]

[1]Sorbonne Université, CNRS, Biologie Intégrative des Organismes Marins, BIOM, Observatoire Océanologique, F-66650 Banyuls/Mer, France

Corresponding author*: mathilde.scheifler@gmail.com

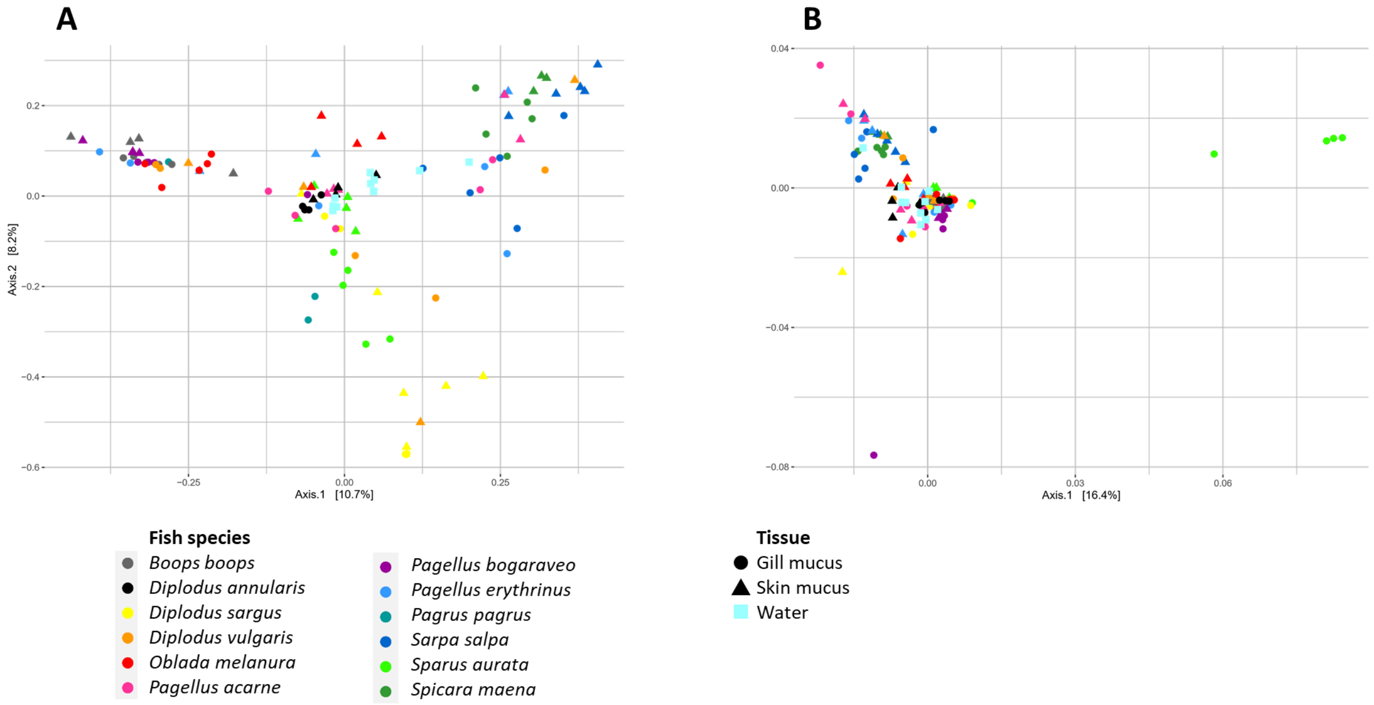

Fig S1. Dissimilarities between bacterial communities of all samples included in this study. PCoA plot representing all fish skin mucus, gill mucus and water bacterial communities based on Bray-Curtis (A) and weighted Unifrac (B) dissimilarity values. Each dot represents one community (water sample or fish individual). Color and shape of dots indicate fish species and community type

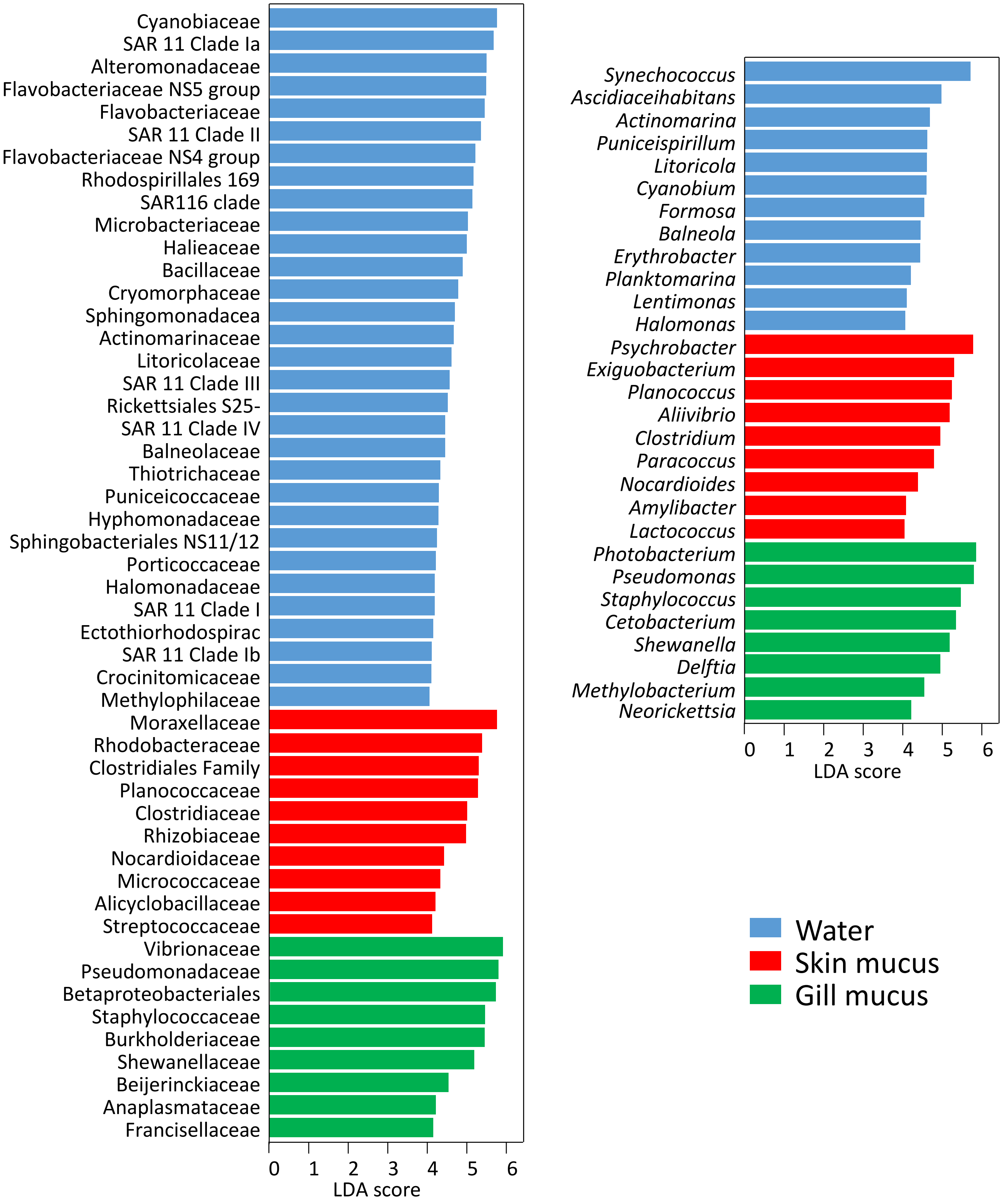

Fig S2. Most contributing taxa to differences between water (blue), skin (red) and gill (green) bacterial communities. (A) Family and (B) genus level. LDA scores were calculated using Linear discriminant analysis Effect Size (LEfSe), only bacterial taxa that raised an LDA score > 4 are shown

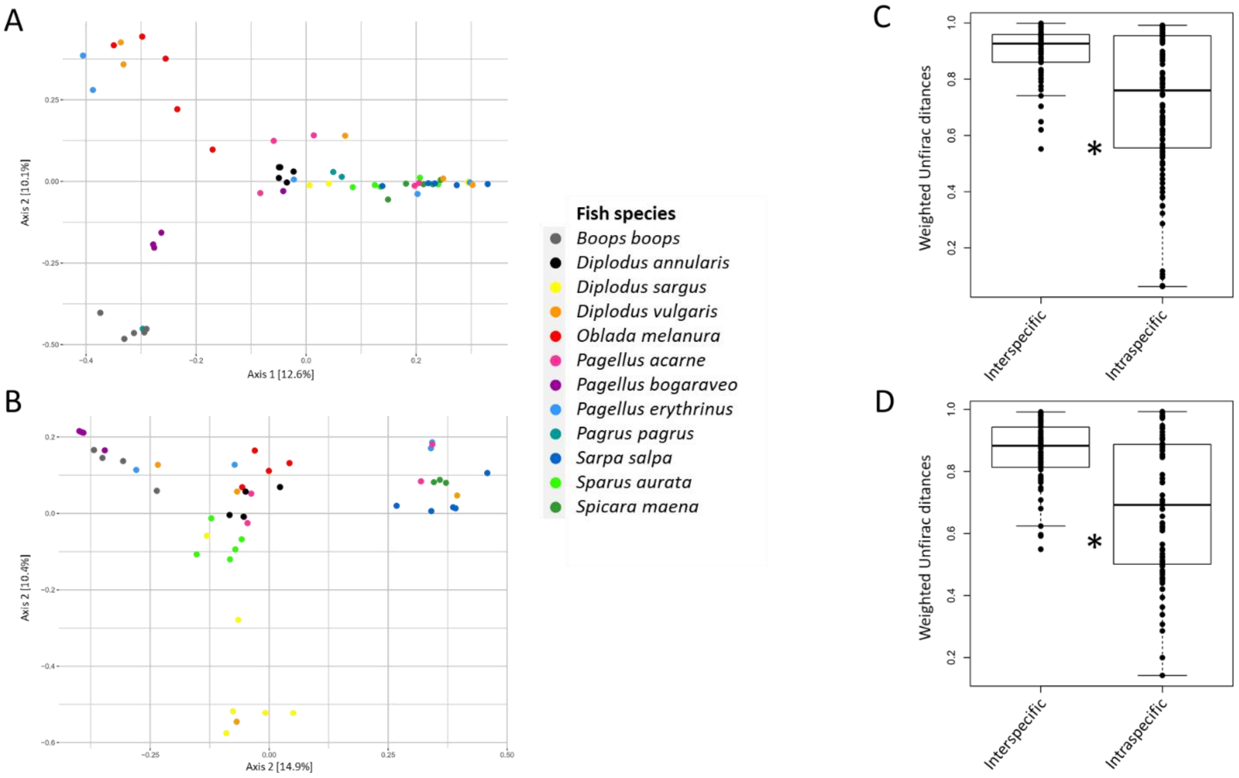

Fig S3. Dissimilarities between bacterial communities based on Bray-Curtis dissimilarity values. PCoA plot representing all fish gill mucus (A) and skin mucus (B) microbiota included in this study**.** Color indicates fish species. Intraspecific and interspecific values based on Bray-Curtis dissimilarity within gill mucus (C) and skin mucus (D) microbiota

*: Significant differences between intraspecific and interspecific values (Kruskal-Wallis test, $p < 0.05$)

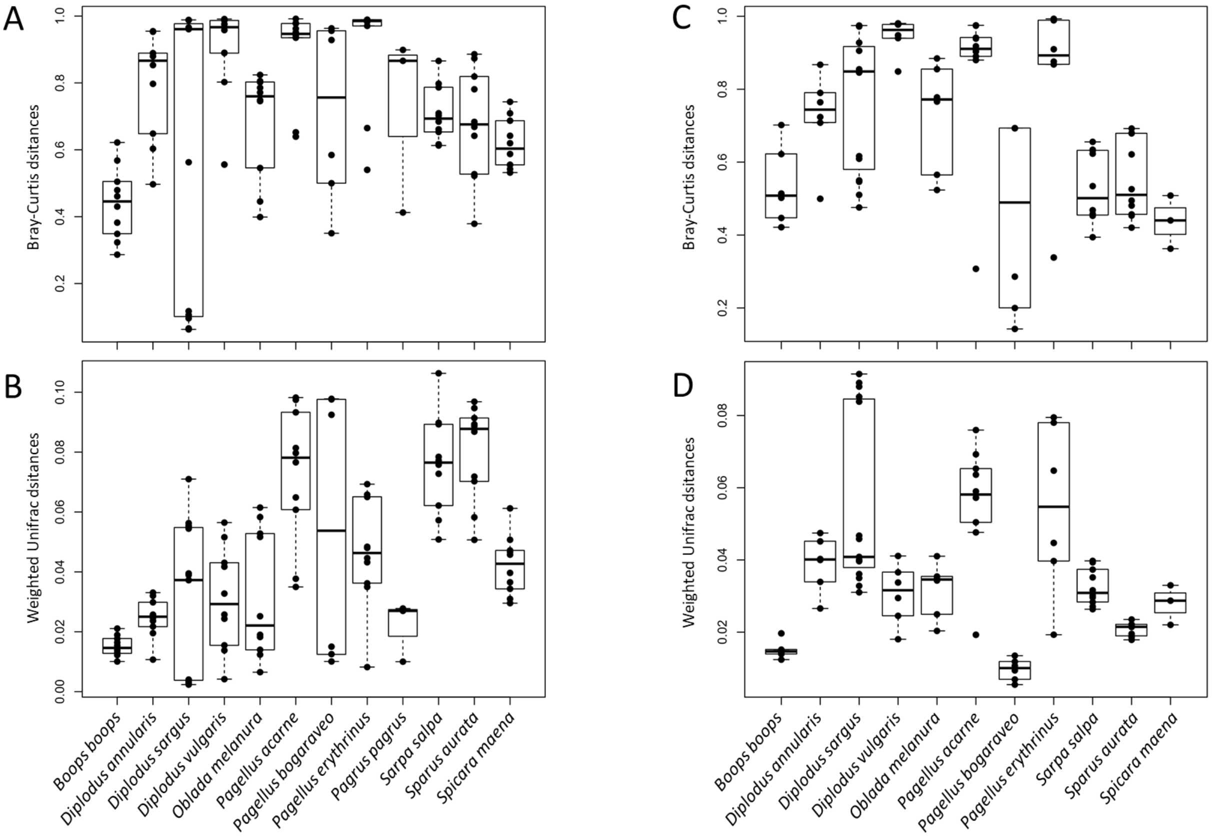

Fig S4. Intraspecific values within each fish species. Bray-Curtis and weighted Unifrac dissimilarities within gill mucus (A, B respectively) and skin mucus (C, D respectively) microbiota

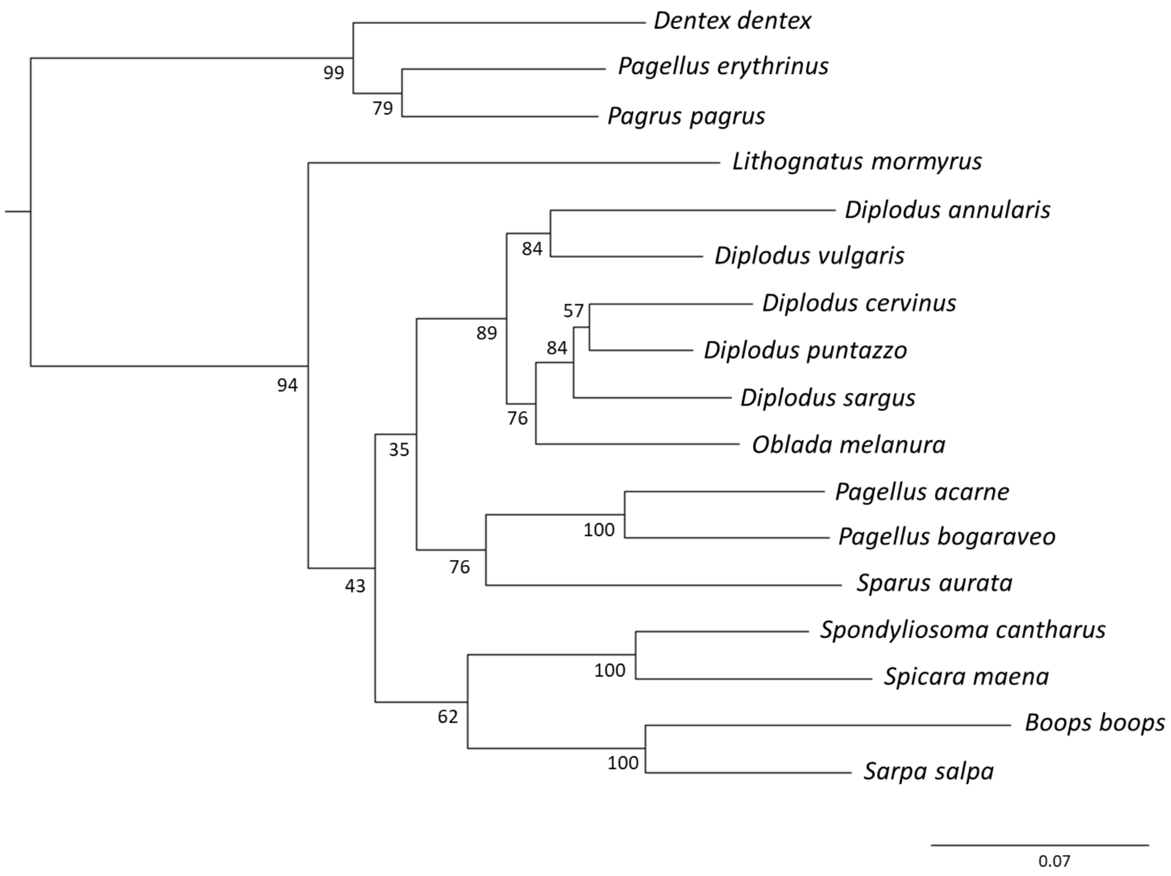

Fig S5. Phylogenetic tree of sparids included in this study. This tree is inferred by maximum likelihood using partitioned dataset (rhodopsin, cytochrome b, cytochrome c oxidase subunit 1 and 16S) conducted with IQ-Tree. Numbers at branches indicate support values, based on 10000 ultrafast bootstrap replicates. *Dicentrarchus labrax* is used as outgroup (Chiba et al. 2009)

Lamellodiscus species	Bacterial genus	WO	W
Furnestinia echeneis	*Cetobacterium*	-0,296	
	Megaira	0,434	
	Photobacterium	-0,379	
	Piscichlamydia	0,360	
	Shewanella	-0,338	
	Similichlamydia	0,467	
L. drummondi	*Staphylococcus*	0,284	
	Vagococcus	0,498	
L. elegans	*Anderseniella*		-0,309
	Aquibacter		-0,289
	Arcobacter		-0,281
	Aureimonas		-0,309
	Bradymonas		-0,385
	Carnobacterium		0,290
	Cetobacterium	-0,353	-0,357
	Clostridium		-0,275
	Cobetia		-0,385
	Erythrobacter		-0,306
	Fluviicola		-0,318
	Formosa		-0,341
	Gramella		-0,342
	Halioglobus	-0,339	-0,301
	Ilumatobacter	-0,290	
	Lentilitoribacter		-0,285
	Lentimonas		-0,341
	Luteolibacter		-0,409
	Lutimonas		-0,368
	Lysinibacillus		-0,271
	Maribacter		-0,344
	Marinobacter		-0,385
	Marixanthomonas		-0,318
	Megaira		-0,274
	Mesonia		-0,363
	Microbacterium		-0,271
	Moritella	-0,285	-0,363
	Parafrigoribacterium		-0,295
	Paraglaciecola		-0,318
	Parasphingopyxis		-0,268
	Persicirhabdus		-0,387
	Planococcus		-0,320
	Propionigenium	-0,493	-0,458
	Providencia		0,281
	Pseudoalteromonas		-0,279
	Psychrilyobacter		-0,407
	Rheinheimera		-0,295
	Rhodopirellula		-0,269
	Roseibacillus		-0,277
	Rubritalea		-0,326
	Salegentibacter		-0,363
	Salinimicrobium		-0,297
	Sulfitobacter		-0,352
	Synechococcus		-0,338
	Thioalkalivibrio		-0,385
	Ulvibacter		-0,341
	Vibrio	-0,441	-0,416
	Vitellibacter		-0,318
L. parisi	*Aliivibrio*	-0,300	
	Blastopirellula	0,398	
	Haloferula	0,365	
	Megaira	0,288	
	Neorickettsia	0,488	
	Photobacterium	-0,281	
	Piscichlamydia	0,397	
	Pseudahrensia	0,390	
	Rhodopirellula	0,462	
	Roseibacillus	0,447	
	Rubripirellula	0,354	
	Rubritalea	0,405	
	Similichlamydia	0,442	
	Streptomyces	0,282	
L. virgula	*Staphylococcus*	0,284	
	Vagococcus	0,497	

Lamellodiscus species	Bacterial genus	WO	W
L. ergensi	*Aquibacter*		-0,340
	Aureimonas		-0,327
	Blastopirellula		-0,376
	Bradymonas		-0,292
	Cetobacterium	-0,344	-0,394
	Clostridium	-0,331	-0,462
	Cobetia		-0,292
	Cyanobium		-0,309
	Enterobacter	0,443	0,398
	Erythrobacter		-0,401
	Gramella		-0,380
	Ilumatobacter	-0,285	
	Maribacter		-0,324
	Marinobacter		-0,292
	Mesonia		-0,276
	Methyloceanibacter		-0,281
	Moritella		-0,276
	Nitrosomonas		-0,276
	Nocardioides		-0,410
	Peredibacter		-0,270
	Photobacterium		-0,374
	Propionigenium	-0,358	-0,368
	Pseudahrensia		-0,341
	Pseudoalteromonas		-0,318
	Pseudomonas	0,334	0,418
	Psychrilyobacter		-0,463
	Psychrobacter		-0,291
	Rhodopirellula		-0,371
	Romboutsia		-0,361
	Rubripirellula		-0,273
	Rubritalea		-0,280
	Salegentibacter		-0,276
	Shewanella	-0,383	-0,379
	Stenotrophomonas		0,385
	Sulfitobacter		-0,304
	Synechococcus		-0,453
	Thioalkalivibrio		-0,292
	Tumebacillus		-0,286
	Vibrio	-0,504	-0,556
L. ignoratus	*Aeromicrobium*		-0,271
	Aliivibrio	-0,428	-0,502
	Amaricoccus		0,412
	Arenibacter		-0,306
	Bradymonas		-0,290
	Bradyrhizobium		0,284
	Coxiella		0,340
	Enhydrobacter		0,295
	Enterovibrio	-0,285	-0,295
	Erythrobacter		-0,271
	Marinobacter		-0,385
	Marixanthomonas		-0,319
	Mesonia		-0,363
	Moritella	-0,285	
	Neorickettsia	0,427	0,328
	Peredibacter		-0,302
	Photobacterium	-0,367	-0,461
	Propionigenium	-0,493	
	Pseudoalteromonas		-0,333
	Pseudomonas	0,465	0,373
	Psychrilyobacter		-0,388
	Salegentibacter		-0,363
	Shewanella	-0,505	-0,410
	Similichlamydia	0,380	0,268
	Streptococcus		0,287
	Vibrio	-0,608	-0,608
	Vitellibacter		-0,319
	Winogradskyella		-0,363

- > 0.45
- 0.3 – 0.45
- 0 – 0.3
- -0.3 – 0
- -0.45 – -0.3
- < -0.45

Fig S6. Significant Spearman correlation coefficients between the abundances of *Lamellodiscus* species and bacterial genus in fish gills. Comparison computed with (W) and without (WO) considering sequences in water samples. Bacterial genera present in the two categories are in bold**.** The color of each cell represents positive (blue) and negative (red) correlations coefficients

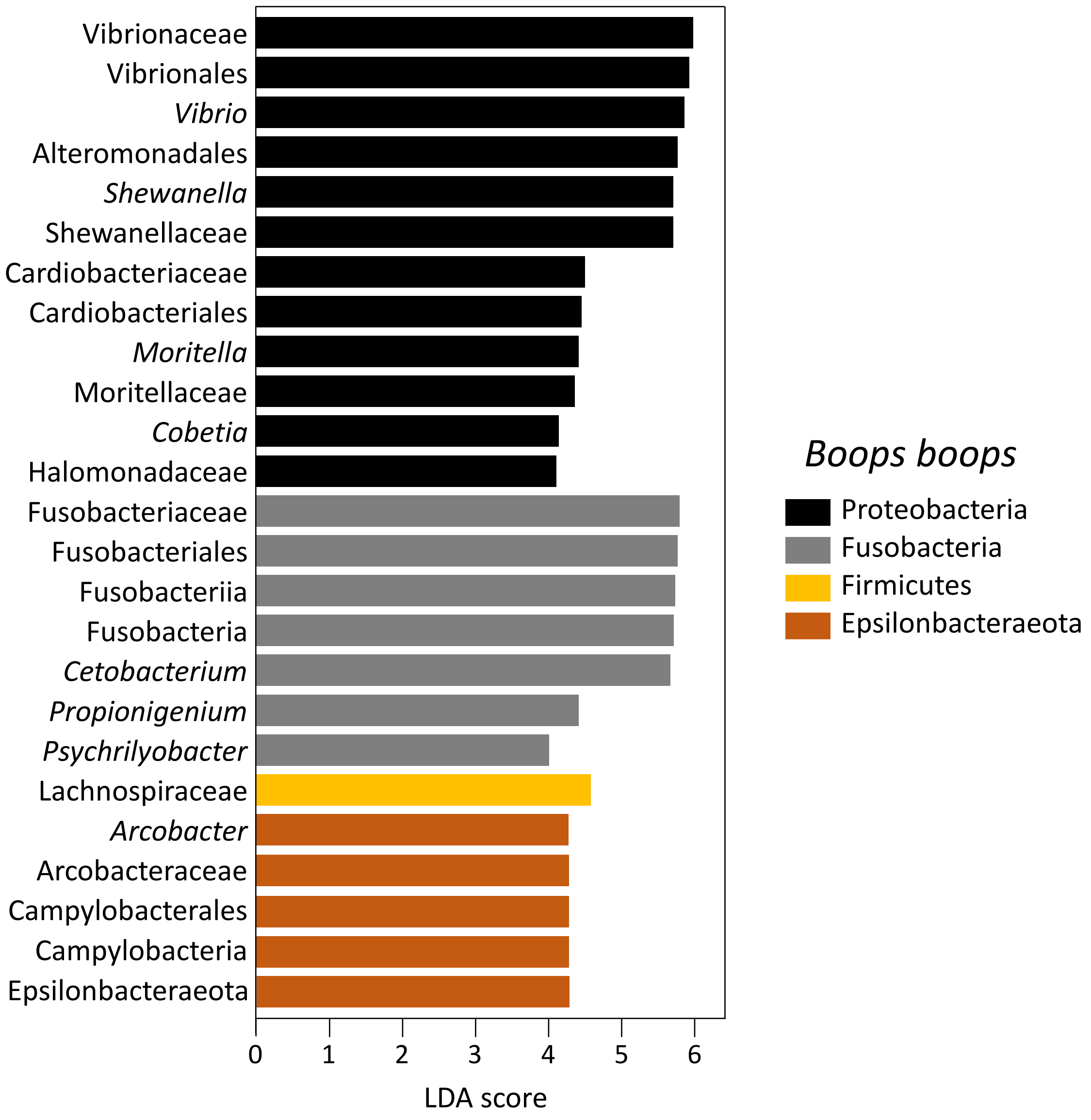

Fig S7. Taxa significantly more associated to *Boops boops*' gill mucus microbiota. LDA scores were calculated using Linear discriminant analysis Effect Size (LEfSe). Only bacterial taxa that raised an LDA score > 4 are shown

Table S1. Specificity of *Lamellodiscus* species within Sparids. *Furnestinia echeneis* was added to the *Lamellodiscus* genus [58]

Lamellodiscus spp.	Sparid hosts	Number of hosts
L. abbreviatus	*Diplodus sargus*	1
L. baeri	*Pagrus pagrus*	1
L. bidens	*Diplodus puntazzo*	1
L. coronatus	*Diplodus annularis, Diplodus cervinus, Diplodus sargus*	3
L. drummondi	*Pagellus acarne*	1
L. diplodi	*Diplodus sargus, Diplodus vulgaris*	2
L. elegans	*Diplodus annularis, Diplodus sargus, Diplodus vulgaris, Oblada melanura, Spondyliosoma cantharus*	5
L. ergensi	*Diplodus annularis, Diplodus puntazzo, Diplodus sargus, Diplodus vulgaris*	4
L. erythrini	*Pagellus erythrinus*	1
L. falcus	*Diplodus sargus, Diplodus vulgaris*	2
L. fraternus	*Diplodus annularis, Diplodus vulgaris*	2
L. furcosus	*Diplodus annularis, Diplodus sargus*	2
L. gracilis	*Diplodus annularis, Diplodus sargus, Oblada melanura*	3
L. gussevi	*Diplodus sargus*	1
L. hilii	*Diplodus puntazzo*	1
L. ignoratus	*Diplodus annularis, Diplodus puntazzo, Diplodus sargus, Diplodus vulgaris, Lithognathus mormyrus, Sarpa salpa*	6
L. impervius	*Diplodus puntazzo*	1
L. knoepffleri	*Spondyliosoma cantharus, Spicara maena, Spicara smaris, Spicara flexuosa*	4
L. mirandus	*Diplodus sargus*	1
L. mormyri	*Lithognathus mormyrus*	1
L. neifari	*Diplodus sargus, Diplodus vulgaris*	2
L. obeliae	*Pagellus bogaraveo*	1
L. oliveri	*Diplodus vulgaris*	1
L. parisi	*Sarpa salpa*	1
L. tomentosus	*Diplodus sargus, Diplodus vulgaris*	2
L. verberis	*Lithognathus mormyrus*	1
L. virgula	*Pagellus acarne*	1

F. echeneis	*Sparus aurata*	1

Table S2. Ecological traits of fish species tested in this study

				Living in … environment			
Fish species	Position water column	Schooling behavior	Diet	Sandy	Muddy	Rocky	Grassy
Boops boops	Pelagic	Group	Omnivorous	Yes	Yes	Yes	Yes
Dentex dentex	Sea bottom	Solitary	Carnivorous	No	No	Yes	No
Diplodus annularis	Sea bottom	Group	Carnivorous	Yes	No	No	Yes
Diplodus puntazzo	Sea bottom	Group	Omnivorous	No	No	Yes	No
Diplodus sargus	Sea bottom	Group	Carnivorous	Yes	No	Yes	No
Diplodus vulgaris	Sea bottom	Group	Carnivorous	Yes	No	Yes	No
Oblada melanura	Pelagic	Group	Omnivorous	No	No	Yes	Yes
Pagellus acarne	Sea bottom	Group	Omnivorous	Yes	Yes	No	Yes
Pagellus bogaraveo	Sea bottom	Group	Omnivorous	Yes	Yes	Yes	No
Pagellus erythrinus	Sea bottom	Group	Omnivorous	Yes	Yes	Yes	No
Pagrus pagrus	Sea bottom	Solitary	Carnivorous	Yes	No	Yes	No
Sarpa salpa	Sea bottom	Group	Herbivorous	Yes	Yes	Yes	Yes
Sparus aurata	Sea bottom	Group	Carnivorous	Yes	No	Yes	Yes
Spicara maena	Pelagic	Group	Omnivorous	No	Yes	Yes	Yes
Spondyliosoma cantharus	Sea bottom	Group	Omnivorous	Yes	No	Yes	Yes

Table S3. **Abiotic factors for each sampling date measured by the SOMLIT (https://www.somlit.fr/)**

		Temperature ºC	Salinity (PSU)	Oxygen (ml/l)	NH_4 (µmol/l)	NO_3 (µmol/l)	NO_2 (µmol/l)	PO_4 (µmol/l)	$SiOH_4$ (µmol/l)
2017	Jun. 21	20.92	37.896	5.29	8.14	0	0.08	0.01	0.01
2018	Apr. 18	13.476	35.498	6.1	7.95	0.291	4.84	0.32	0.09
	Jun. 26	20.402	37.418	5.27	8.02	0.022	1.26	0.16	0.03
	Jul. 13	22.097	38.155	5.17	7.99	0.036	0.02	0.01	0.01
	Sept. 4	20.929	38.096	5.11	-	0.01	0.06	0.01	0.02
	Oct. 5	18.761	38.167	5.25	8.17	0.067	0.02	0.01	0.01
2019	Mar. 22	13.102	38.017	5.84	8.17	0.036	1.2	0.18	0.02
	Mar. 28	12.556	38.149	5.95	7.91	0.087	0.7	0.27	0.05
	May 10	14.508	38.043	5.85	8.13	0.029	0.05	0.02	0.04
	Jun. 6	17.168	38.062	5.67	7.94	0.009	0.02	0.02	0.06
	Jun. 28	20.839	37.538	5.46	7.99	0.057	0.02	0.01	0.04
	Jul. 8	25.265	36.542	4.81	8.04	0.041	0.02	0.01	0.03
	Jul. 10	25.265	36.542	4.81	8.04	0.041	0.02	0.01	0.03

Table S4. Non-significant and significant (in bold) pairwise comparisons between fish species skin or gill mucus diversity. Post hoc Conover-Iman test significant if *p* < alpha/2 = 0.025

	Faith's phylogenetic index		Shannon index	
Comparison	Gills	Skin	Gills	Skin
Boops boops - Diplodus annularis	**0.015**	0.049	0.042	0.465
Boops boops - Diplodus sargus	0.241	0.195	**0.003**	0.465
Boops boops - Diplodus vulgaris	0.091	0.359	0.273	0.492
Boops boops - Oblada melanura	0.121	0.199	0.295	0.488
Boops boops - Pagellus acarne	0.135	0.354	0.371	0.500
Boops boops - Pagellus bogaraveo	0.184	**0.013**	0.214	0.494
Boops boops - Pagellus erythrinus	0.230	0.159	0.461	0.214
Boops boops - Pagrus pagrus	0.257	-	0.468	-
Boops boops - Sarpa salpa	**0.000**	**0.000**	0.055	**0.004**
Boops boops - Sparus aurata	0.032	0.055	0.209	0.070
Boops boops - Spicara maena	**0.000**	**0.001**	**0.003**	**0.013**
Diplodus annularis - Diplodus sargus	0.076	**0.003**	0.211	0.499
Diplodus annularis - Diplodus vulgaris	**0.000**	**0.017**	0.165	0.479
Diplodus annularis - Oblada melanura	**0.000**	**0.004**	0.155	0.487
Diplodus annularis - Pagellus acarne	**0.001**	**0.013**	0.118	0.465
Diplodus annularis - Pagellus bogaraveo	0.162	0.324	0.244	0.460
Diplodus annularis - Pagellus erythrinus	**0.001**	**0.003**	0.067	0.179
Diplodus annularis - Pagrus pagrus	**0.004**	-	0.048	-
Diplodus annularis - Sarpa salpa	**0.000**	**0.000**	**0.000**	**0.003**
Diplodus annularis - Sparus aurata	**0.000**	**0.000**	0.216	0.048
Diplodus annularis - Spicara maena	**0.000**	**0.000**	**0.000**	**0.009**
Diplodus sargus - Diplodus vulgaris	**0.013**	0.347	**0.021**	0.480
Diplodus sargus - Oblada melanura	**0.018**	0.476	**0.019**	0.485
Diplodus sargus - Pagellus acarne	**0.021**	0.336	**0.012**	0.465
Diplodus sargus - Pagellus bogaraveo	0.412	**0.000**	0.055	0.459
Diplodus sargus - Pagellus erythrinus	0.049	0.421	**0.005**	0.154
Diplodus sargus - Pagrus pagrus	0.079	-	**0.005**	-
Diplodus sargus - Sarpa salpa	**0.000**	**0.001**	**0.000**	**0.001**
Diplodus sargus - Sparus aurata	**0.003**	0.243	0.038	0.031
Diplodus sargus - Spicara maena	**0.000**	**0.008**	**0.000**	**0.005**
Diplodus vulgaris - Oblada melanura	0.446	0.340	0.496	0.495
Diplodus vulgaris - Pagellus acarne	0.423	0.497	0.456	0.495
Diplodus vulgaris - Pagellus bogaraveo	**0.011**	**0.004**	0.454	0.487
Diplodus vulgaris - Pagellus erythrinus	0.324	0.289	0.361	0.207
Diplodus vulgaris - Pagrus pagrus	0.354	-	0.243	-
Diplodus vulgaris - Sarpa salpa	**0.001**	**0.001**	**0.010**	**0.004**
Diplodus vulgaris - Sparus aurata	0.348	0.135	0.461	0.064
Diplodus vulgaris - Spicara maena	**0.001**	**0.004**	**0.000**	**0.011**
Oblada melanura - Pagellus acarne	0.480	0.328	0.466	0.490
Oblada melanura - Pagellus bogaraveo	**0.016**	**0.001**	0.440	0.484
Oblada melanura - Pagellus erythrinus	0.368	0.447	0.378	0.202
Oblada melanura - Pagrus pagrus	0.397	-	0.257	-
Oblada melanura - Sarpa salpa	**0.001**	**0.004**	**0.011**	**0.004**
Oblada melanura - Sparus aurata	0.294	0.288	0.444	0.060
Oblada melanura - Spicara maena	**0.001**	**0.015**	**0.000**	**0.011**

Pagellus acarne - Pagellus bogaraveo	**0.018**	**0.003**	0.372	0.497
Pagellus acarne - Pagellus erythrinus	0.391	0.275	0.449	0.206
Pagellus acarne - Pagrus pagrus	0.413	-	0.317	-
Pagellus acarne - Sarpa salpa	**0.000**	**0.000**	**0.017**	**0.003**
Pagellus acarne - Sparus aurata	0.275	0.118	0.373	0.057
Pagellus acarne - Spicara maena	**0.001**	**0.003**	**0.001**	**0.010**
Pagellus bogaraveo - Pagellus erythrinus	0.040	**0.001**	0.275	0.228
Pagellus bogaraveo - Pagrus pagrus	0.060	-	0.201	-
Pagellus bogaraveo - Sarpa salpa	**0.000**	**0.000**	**0.007**	**0.005**
Pagellus bogaraveo - Sparus aurata	**0.003**	**0.000**	0.494	0.077
Pagellus bogaraveo - Spicara maena	**0.000**	**0.000**	**0.000**	**0.014**
Pagellus erythrinus - Pagrus pagrus	0.494	-	0.405	-
Pagellus erythrinus - Sarpa salpa	**0.000**	**0.006**	0.033	0.048
Pagellus erythrinus - Sparus aurata	0.172	0.344	0.272	0.302
Pagellus erythrinus - Spicara maena	**0.000**	**0.020**	**0.002**	0.094
Pagrus pagrus - Sarpa salpa	**0.001**	-	0.131	-
Pagrus pagrus - Sparus aurata	0.218	-	0.199	-
Pagrus pagrus - Spicara maena	**0.001**	-	**0.013**	-
Sarpa salpa - Sparus aurata	**0.004**	**0.016**	**0.005**	0.150
Sarpa salpa - Spicara maena	0.470	0.439	0.154	0.485
Sparus aurata - Spicara maena	**0.005**	0.049	**0.000**	0.207

Table S5. Non-significant (in bold) and significant pairwise comparisons between fish species within skin or gill mucus. Based on Pairwise PERMANOVAs, non-significant if $p > 0.05$

Skin mucus Bray-Curtis	
Associations	P-value
Boops boops vs *Diplodus annularis*	0.030
Boops boops vs *Diplodus vulgaris*	0.029
Boops boops vs *Oblada melanura*	0.028
Boops boops vs *Pagellus acarne*	0.008
Boops boops vs *Pagellus bogaraveo*	0.027
Boops boops vs *Pagellus erythrinus*	0.029
Boops boops vs *Sarpa salpa*	0.007
Diplodus sargus vs *Boops boops*	0.005
Diplodus sargus vs *Diplodus annularis*	0.004
Diplodus sargus vs *Diplodus vulgaris*	**0.077**
Diplodus sargus vs *Oblada melanura*	0.005
Diplodus sargus vs *Pagellus acarne*	0.005
Diplodus sargus vs *Pagellus bogaraveo*	0.005
Diplodus sargus vs *Pagellus erythrinus*	0.009
Diplodus sargus vs *Sarpa salpa*	0.002
Diplodus sargus vs *Spicara maena*	0.011
Diplodus vulgaris vs *Diplodus annularis*	0.030
Diplodus vulgaris vs *Oblada melanura*	**0.088**
Oblada melanura vs *Diplodus annularis*	0.029
Pagellus acarne vs *Diplodus annularis*	0.017
Pagellus acarne vs *Diplodus vulgaris*	**0.259**
Pagellus acarne vs *Oblada melanura*	**0.115**
Pagellus acarne vs *Pagellus bogaraveo*	0.008
Pagellus bogaraveo vs *Diplodus annularis*	0.030
Pagellus bogaraveo vs *Diplodus vulgaris*	0.028
Pagellus bogaraveo vs *Oblada melanura*	0.029
Pagellus erythrinus vs *Diplodus annularis*	0.030
Pagellus erythrinus vs *Diplodus vulgaris*	**0.162**
Pagellus erythrinus vs *Oblada melanura*	**0.263**
Pagellus erythrinus vs *Pagellus acarne*	**0.621**
Pagellus erythrinus vs *Pagellus bogaraveo*	0.025
Sarpa salpa vs *Diplodus annularis*	0.008
Sarpa salpa vs *Diplodus vulgaris*	0.040
Sarpa salpa vs *Oblada melanura*	0.009
Sarpa salpa vs *Pagellus acarne*	0.009
Sarpa salpa vs *Pagellus bogaraveo*	0.009
Sarpa salpa vs *Pagellus erythrinus*	0.009
Sparus aurata vs *Boops boops*	0.007
Sparus aurata vs *Diplodus annularis*	0.010
Sparus aurata vs *Diplodus sargus*	0.002
Sparus aurata vs *Diplodus vulgaris*	0.009
Sparus aurata vs *Oblada melanura*	0.008
Sparus aurata vs *Pagellus acarne*	0.008
Sparus aurata vs *Pagellus bogaraveo*	0.009
Sparus aurata vs *Pagellus erythrinus*	0.007

Gill mucus Bray-Curtis	
Associations	P-value
Boops boops vs *Diplodus annularis*	0.008
Boops boops vs *Diplodus vulgaris*	0.009
Boops boops vs *Oblada melanura*	0.01
Boops boops vs *Pagellus acarne*	0.008
Boops boops vs *Pagellus bogaraveo*	0.008
Boops boops vs *Pagellus erythrinus*	0.008
Boops boops vs *Sarpa salpa*	0.008
Diplodus sargus vs *Boops boops*	0.002
Diplodus sargus vs *Diplodus annularis*	0.002
Diplodus sargus vs *Diplodus vulgaris*	0.024
Diplodus sargus vs *Oblada melanura*	0.002
Diplodus sargus vs *Pagellus acarne*	0.019
Diplodus sargus vs *Pagellus bogaraveo*	0.008
Diplodus sargus vs *Pagellus erythrinus*	0.02
Diplodus sargus vs *Pagrus pagrus*	0.023
Diplodus sargus vs *Sarpa salpa*	0.003
Diplodus sargus vs *Spicara maena*	0.002
Diplodus vulgaris vs *Diplodus annularis*	0.008
Diplodus vulgaris vs *Oblada melanura*	**0.283**
Oblada melanura vs *Diplodus annularis*	0.007
Pagellus acarne vs *Diplodus annularis*	0.009
Pagellus acarne vs *Diplodus vulgaris*	**0.252**
Pagellus acarne vs *Oblada melanura*	0.009
Pagellus acarne vs *Pagellus bogaraveo*	0.023
Pagellus bogaraveo vs *Diplodus annularis*	0.008
Pagellus bogaraveo vs *Diplodus vulgaris*	0.01
Pagellus bogaraveo vs *Oblada melanura*	0.018
Pagellus erythrinus vs *Diplodus annularis*	0.009
Pagellus erythrinus vs *Diplodus vulgaris*	**0.844**
Pagellus erythrinus vs *Oblada melanura*	**0.188**
Pagellus erythrinus vs *Pagellus acarne*	**0.408**
Pagellus erythrinus vs *Pagellus bogaraveo*	0.023
Pagrus pagrus vs *Boops boops*	0.037
Pagrus pagrus vs *Diplodus annularis*	0.019
Pagrus pagrus vs *Diplodus vulgaris*	**0.106**
Pagrus pagrus vs *Oblada melanura*	0.019
Pagrus pagrus vs *Pagellus acarne*	**0.07**
Pagrus pagrus vs *Pagellus bogaraveo*	**0.086**
Pagrus pagrus vs *Pagellus erythrinus*	**0.16**
Pagrus pagrus vs *Sarpa salpa*	0.02
Sarpa salpa vs *Diplodus annularis*	0.007
Sarpa salpa vs *Diplodus vulgaris*	0.008
Sarpa salpa vs *Oblada melanura*	0.008
Sarpa salpa vs *Pagellus acarne*	0.007
Sarpa salpa vs *Pagellus bogaraveo*	0.009

Sparus aurata vs *Sarpa salpa*	0.008
Sparus aurata vs *Spicara maena*	0.017
Spicara maena vs *Boops boops*	0.029
Spicara maena vs *Diplodus annularis*	0.029
Spicara maena vs *Diplodus vulgaris*	**0.057**
Spicara maena vs *Oblada melanura*	0.029
Spicara maena vs *Pagellus acarne*	0.035
Spicara maena vs *Pagellus bogaraveo*	0.029
Spicara maena vs *Pagellus erythrinus*	0.029
Spicara maena vs *Sarpa salpa*	0.017

Sarpa salpa vs *Pagellus erythrinus*	0.008
Sparus aurata vs *Boops boops*	0.007
Sparus aurata vs *Diplodus annularis*	0.007
Sparus aurata vs *Diplodus sargus*	0.004
Sparus aurata vs *Diplodus vulgaris*	0.007
Sparus aurata vs *Oblada melanura*	0.008
Sparus aurata vs *Pagellus acarne*	0.008
Sparus aurata vs *Pagellus bogaraveo*	0.009
Sparus aurata vs *Pagellus erythrinus*	0.008
Sparus aurata vs *Pagrus pagrus*	0.018
Sparus aurata vs *Sarpa salpa*	0.009
Sparus aurata vs *Spicara maena*	0.008
Spicara maena vs *Boops boops*	0.007
Spicara maena vs *Diplodus annularis*	0.007
Spicara maena vs *Diplodus vulgaris*	0.008
Spicara maena vs *Oblada melanura*	0.01
Spicara maena vs *Pagellus acarne*	0.008
Spicara maena vs *Pagellus bogaraveo*	0.008
Spicara maena vs *Pagellus erythrinus*	0.009
Spicara maena vs *Pagrus pagrus*	0.018
Spicara maena vs *Sarpa salpa*	0.007

Skin mucus Weighted Unifrac	
Associations	P-value
Boops boops vs *Diplodus annularis*	0.028
Boops boops vs *Diplodus vulgaris*	0.028
Boops boops vs *Oblada melanura*	0.029
Boops boops vs *Pagellus acarne*	**0.337**
Boops boops vs *Pagellus bogaraveo*	0.027
Boops boops vs *Pagellus erythrinus*	**0.233**
Boops boops vs *Sarpa salpa*	0.008
Diplodus sargus vs *Boops boops*	**0.179**
Diplodus sargus vs *Diplodus annularis*	0.005
Diplodus sargus vs *Diplodus vulgaris*	**0.787**
Diplodus sargus vs *Oblada melanura*	0.009
Diplodus sargus vs *Pagellus acarne*	0.002
Diplodus sargus vs *Pagellus bogaraveo*	**0.073**
Diplodus sargus vs *Pagellus erythrinus*	0.005
Diplodus sargus vs *Sarpa salpa*	0.003
Diplodus sargus vs *Spicara maena*	0.014
Diplodus vulgaris vs *Diplodus annularis*	0.027
Diplodus vulgaris vs *Oblada melanura*	**0.089**
Oblada melanura vs *Diplodus annularis*	0.029
Pagellus acarne vs *Diplodus annularis*	0.032
Pagellus acarne vs *Diplodus vulgaris*	**0.778**
Pagellus acarne vs *Oblada melanura*	**0.47**
Pagellus acarne vs *Pagellus bogaraveo*	**0.056**
Pagellus bogaraveo vs *Diplodus annularis*	0.027
Pagellus bogaraveo vs *Diplodus vulgaris*	0.029
Pagellus bogaraveo vs *Oblada melanura*	0.029

Gill mucus Bray-Curtis Weighted Unifrac	
Associations	P-value
Boops boops vs *Diplodus annularis*	0.008
Boops boops vs *Diplodus vulgaris*	0.009
Boops boops vs *Oblada melanura*	0.009
Boops boops vs *Pagellus acarne*	**0.644**
Boops boops vs *Pagellus bogaraveo*	0.009
Boops boops vs *Pagellus erythrinus*	0.008
Boops boops vs *Sarpa salpa*	0.015
Diplodus sargus vs *Boops boops*	0.002
Diplodus sargus vs *Diplodus annularis*	0.002
Diplodus sargus vs *Diplodus vulgaris*	**0.063**
Diplodus sargus vs *Oblada melanura*	0.002
Diplodus sargus vs *Pagellus acarne*	**0.138**
Diplodus sargus vs *Pagellus bogaraveo*	0.005
Diplodus sargus vs *Pagellus erythrinus*	0.004
Diplodus sargus vs *Pagrus pagrus*	**0.365**
Diplodus sargus vs *Sarpa salpa*	0.003
Diplodus sargus vs *Spicara maena*	0.002
Diplodus vulgaris vs *Diplodus annularis*	0.009
Diplodus vulgaris vs *Oblada melanura*	**0.116**
Oblada melanura vs *Diplodus annularis*	0.008
Pagellus acarne vs *Diplodus annularis*	**0.762**
Pagellus acarne vs *Diplodus vulgaris*	**0.858**
Pagellus acarne vs *Oblada melanura*	**0.498**
Pagellus acarne vs *Pagellus bogaraveo*	0.017
Pagellus bogaraveo vs *Diplodus annularis*	0.007
Pagellus bogaraveo vs *Diplodus vulgaris*	0.008

Pagellus erythrinus vs *Diplodus annularis*	0.03
Pagellus erythrinus vs *Diplodus vulgaris*	**0.367**
Pagellus erythrinus vs *Oblada melanura*	**0.345**
Pagellus erythrinus vs *Pagellus acarne*	**0.607**
Pagellus erythrinus vs *Pagellus bogaraveo*	**0.318**
Sarpa salpa vs *Diplodus annularis*	0.008
Sarpa salpa vs *Diplodus vulgaris*	**0.065**
Sarpa salpa vs *Oblada melanura*	0.007
Sarpa salpa vs *Pagellus acarne*	0.009
Sarpa salpa vs *Pagellus bogaraveo*	0.01
Sarpa salpa vs *Pagellus erythrinus*	0.009
Sparus aurata vs *Boops boops*	0.009
Sparus aurata vs *Diplodus annularis*	0.008
Sparus aurata vs *Diplodus sargus*	0.014
Sparus aurata vs *Diplodus vulgaris*	0.009
Sparus aurata vs *Oblada melanura*	0.008
Sparus aurata vs *Pagellus acarne*	**0.211**
Sparus aurata vs *Pagellus bogaraveo*	0.01
Sparus aurata vs *Pagellus erythrinus*	0.048
Sparus aurata vs *Sarpa salpa*	0.007
Sparus aurata vs *Spicara maena*	0.019
Spicara maena vs *Boops boops*	0.029
Spicara maena vs *Diplodus annularis*	0.029
Spicara maena vs *Diplodus vulgaris*	**0.057**
Spicara maena vs *Oblada melanura*	0.029
Spicara maena vs *Pagellus acarne*	**0.054**
Spicara maena vs *Pagellus bogaraveo*	0.029
Spicara maena vs *Pagellus erythrinus*	**0.143**
Spicara maena vs *Sarpa salpa*	0.02

Pagellus bogaraveo vs *Oblada melanura*	0.01
Pagellus erythrinus vs *Diplodus annularis*	**0.082**
Pagellus erythrinus vs *Diplodus vulgaris*	**0.334**
Pagellus erythrinus vs *Oblada melanura*	**0.216**
Pagellus erythrinus vs *Pagellus acarne*	**0.895**
Pagellus erythrinus vs *Pagellus bogaraveo*	0.009
Pagrus pagrus vs *Boops boops*	0.0368
Pagrus pagrus vs *Diplodus annularis*	0.017
Pagrus pagrus vs *Diplodus vulgaris*	**0.352**
Pagrus pagrus vs *Oblada melanura*	**0.321**
Pagrus pagrus vs *Pagellus acarne*	**0.946**
Pagrus pagrus vs *Pagellus bogaraveo*	**0.371**
Pagrus pagrus vs *Pagellus erythrinus*	**0.84**
Pagrus pagrus vs *Sarpa salpa*	**0.22**
Sarpa salpa vs *Diplodus annularis*	0.005
Sarpa salpa vs *Diplodus vulgaris*	0.042
Sarpa salpa vs *Oblada melanura*	0.008
Sarpa salpa vs *Pagellus acarne*	0.008
Sarpa salpa vs *Pagellus bogaraveo*	0.008
Sarpa salpa vs *Pagellus erythrinus*	0.006
Sparus aurata vs *Boops boops*	0.008
Sparus aurata vs *Diplodus annularis*	0.015
Sparus aurata vs *Diplodus sargus*	0.002
Sparus aurata vs *Diplodus vulgaris*	0.024
Sparus aurata vs *Oblada melanura*	0.022
Sparus aurata vs *Pagellus acarne*	0.007
Sparus aurata vs *Pagellus bogaraveo*	0.014
Sparus aurata vs *Pagellus erythrinus*	0.007
Sparus aurata vs *Pagrus pagrus*	**0.073**
Sparus aurata vs *Sarpa salpa*	0.008
Sparus aurata vs *Spicara maena*	0.007
Spicara maena vs *Boops boops*	0.009
Spicara maena vs *Diplodus annularis*	0.008
Spicara maena vs *Diplodus vulgaris*	0.009
Spicara maena vs *Oblada melanura*	0.008
Spicara maena vs *Pagellus acarne*	0.01
Spicara maena vs *Pagellus bogaraveo*	0.008
Spicara maena vs *Pagellus erythrinus*	0.01
Spicara maena vs *Pagrus pagrus*	0.019
Spicara maena vs *Sarpa salpa*	0.007

Table S6. Correlations between interspecific dissimilarities in skin mucus or gill mucus microbiota and host sparid phylogeny. Two methods were used to generate the dissimilarity matrix: random selection of one individual per fish species (A) and averaging the microbial taxa among individuals of each fish species (B). Based on Bray-Curtis (BC) or Weighted Unifrac (WU) dissimilarities

(A) Random selection of one individual per fish species - % of significant p-values (1000 permutations)											
With water sequences				Without water sequences				Core microbiota			
Gill mucus		Skin mucus		Gill mucus		Skin mucus		Gill mucus		Skin mucus	
BC	WU	BC	WU	BC	WU	BC	WU	BC	WU	BC	WU
2%	0%	17.4%	0%	0%	0%	0%	0.6%	15.4%	4.9%	1.4%	1.6%

	(B) Average of microbial taxa among individuals of each fish species											
	With water sequences				Without water sequences				Core microbiota			
	Gill mucus		Skin mucus		Gill mucus		Skin mucus		Gill mucus		Skin mucus	
	BC	WU	BC	WU	BC	WU	BC	WU	BC	WU	BC	WU
P-value	0.88	0.79	0.68	0.51	0.93	0.71	0.79	0.29	0.42	0.75	0.39	0.99

Table S7. Total abundance (i.e. total number of *Lamellodiscus* individuals found on each fish individual). Abundance of each *Lamellodiscus* species and species richness (i.e. number of different *Lamellodiscus* species found on each fish individual) within gills of each fish individual

Fish species	Individual	Total abundance	*L. abbreviatus*	*L. baeri*	*L. coronatus*	*L. diplodi*	*L. drummondi*	*L. elegans*	*L. ergensi*	*L. erythrini*	*L. falcus*	*L. fraternus*	*L. furcosus*	*L. gracilis*	*L. ignoratus*	*L. knoepffleri*	*L. mirandus*	*L. neifari*	*L. parisi*	*L. oliveri*	*L. tomentosus*	*L. virgula*	*Furnestinia echeneis*	*Species richness*
Boops boops	1	0																						0
	2	0																						0
	3	0																						0
	4	0																						0
	5	0																						0
Diplodus annularis	1	35						22	5						8									3
	2	21						12	4						5									3
	3	39						21	3					4	11									4
	4	29						24	3						2									3
	5	43						17	7					11	8									4
Diplodus sargus	1	101	3					31	12						38			11			6			6
	2	74			9			18	9				14	8	16									6
	3	127				7		22	5				21		44		11	17						7
	4	92			8	4		13	4		5		9	4	23		7				15			10
	5	111						25	6		2		11	19	32		16							7
	6	78			14			12	19				4	11	8		10							7
Diplodus vulgaris	1	82						41	4		10	11			14						2			6

	2	50						7	22		13				8									4
	3	54				4		19	16			6			7			2						6
	4	71						27			8	13			6			7		10				6
	5	95				8		32			5	21			19						10			6
Oblada melanura	1	139						113						26										2
	2	146						124						22										2
	3	162						115						47										2
	4	147						103						44										2
	5	121						101						20										2
Pagellus acarne	1	44					28															16		2
	2	67					42															25		2
	3	76					56															20		2
	4	21					8															13		2
	5	38					24															14		2
Pagellus erythrinus	1	12								12														1
	2	16								16														1
	3	31								31														1
	4	9								9														1
	5	15								15														1
Pagrus pagrus	1	115		115																				1
	2	86		86																				1
	3	102		102																				1
Sarpa salpa	1	121													74				47					2
	2	47													26				21					2
	3	80													55				25					2
	4	78													47				31					2
	5	69													51				18					2
Sparus aurata	1	54																					54	1

	2	49																					49	1
	3	18																					18	1
	4	77																					77	1
	5	25																					25	1
Spicara maena	1	22														22								1
	2	11														11								1
	3	7														7								1
	4	28														28								1
	5	6														6								1

Table S8. ***Lamellodiscus*** **species with no significant influence on the composition of gill mucus microbiota.** Performed with Mantel test, $p > 0.05$

Lamellodiscus species	P-value
L. abbreviatus	0.82
L. baeri	0.67
L. coronatus	0.17
L. diplodi	0.57
L. erythrini	0.66
L. falcus	0.36
L. fraternus	0.76
L. furcosus	0.19
L. gracilis	0.53
L. knoepffleri	0.19
L. neifari	0.71
L. oliveri	0.92
L. tomentosus	0.25
